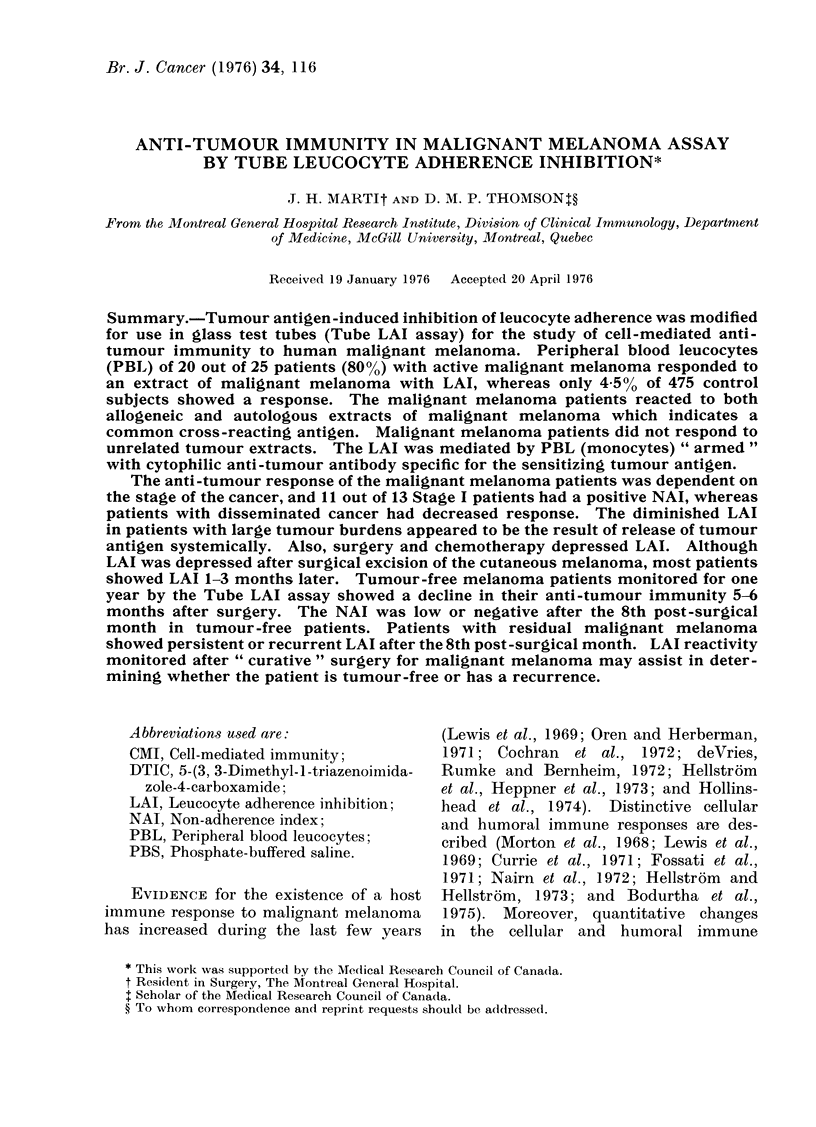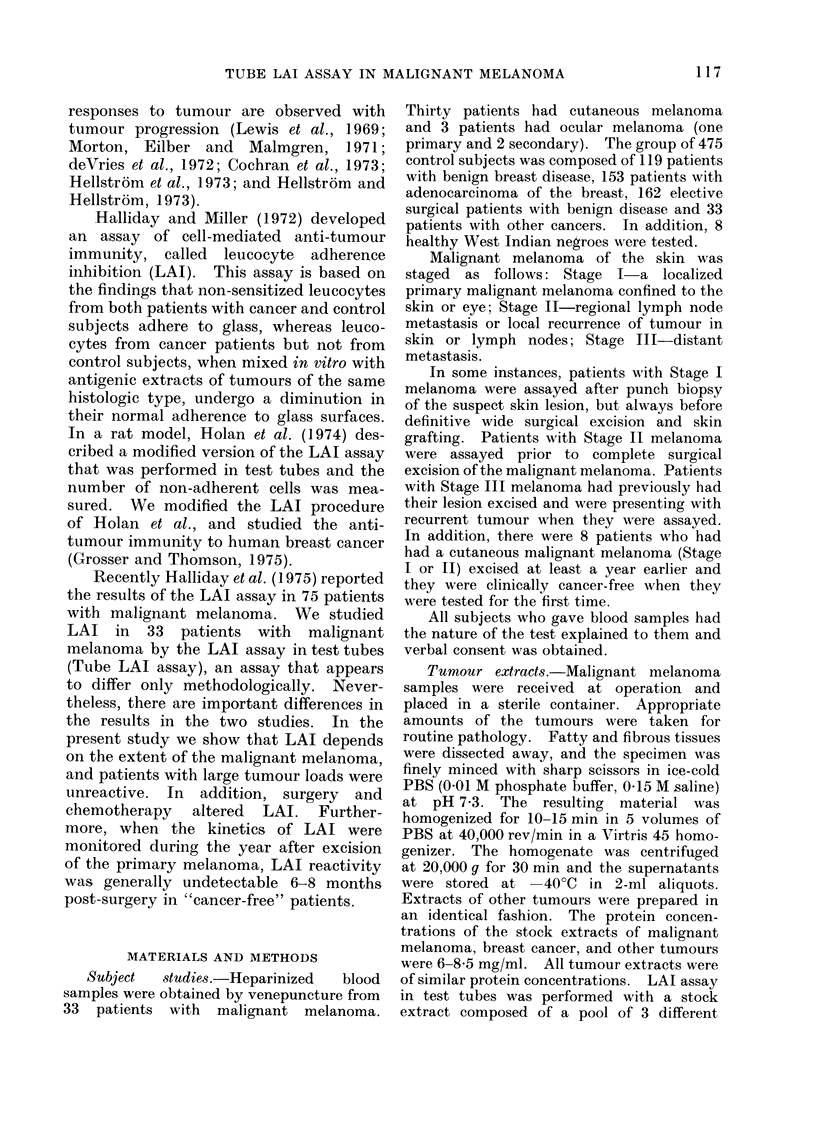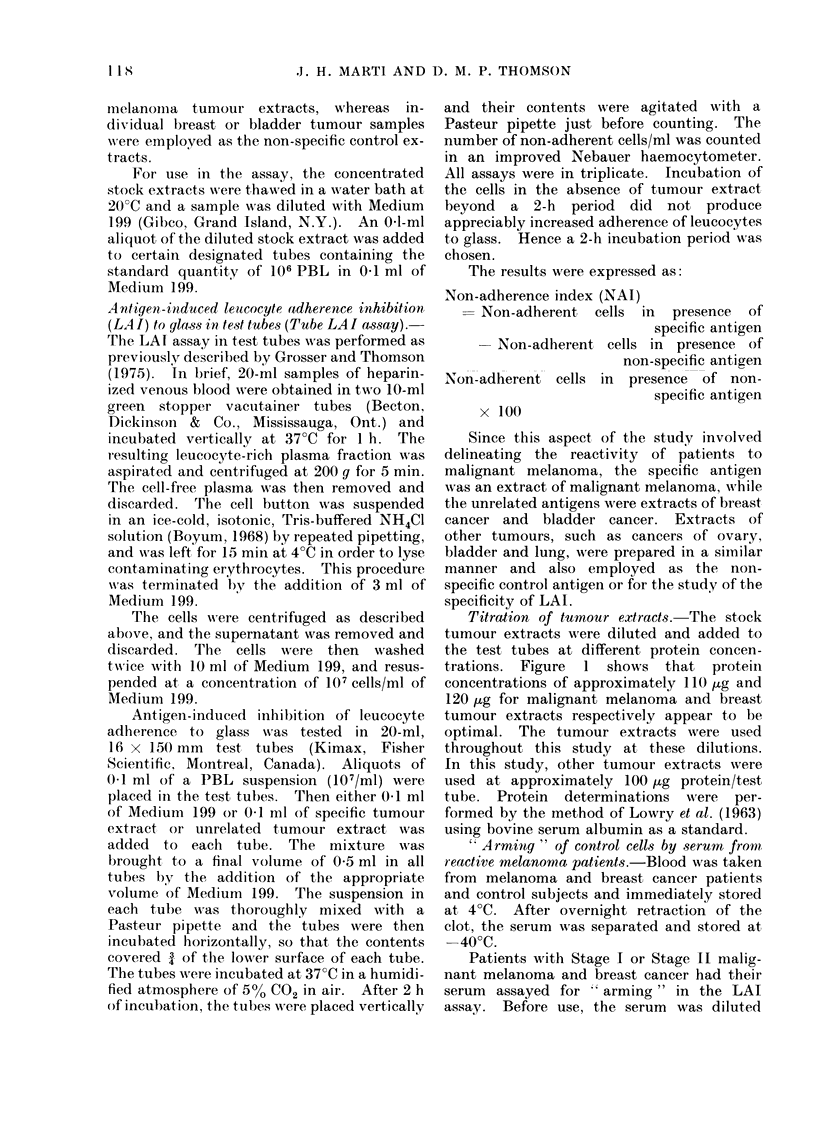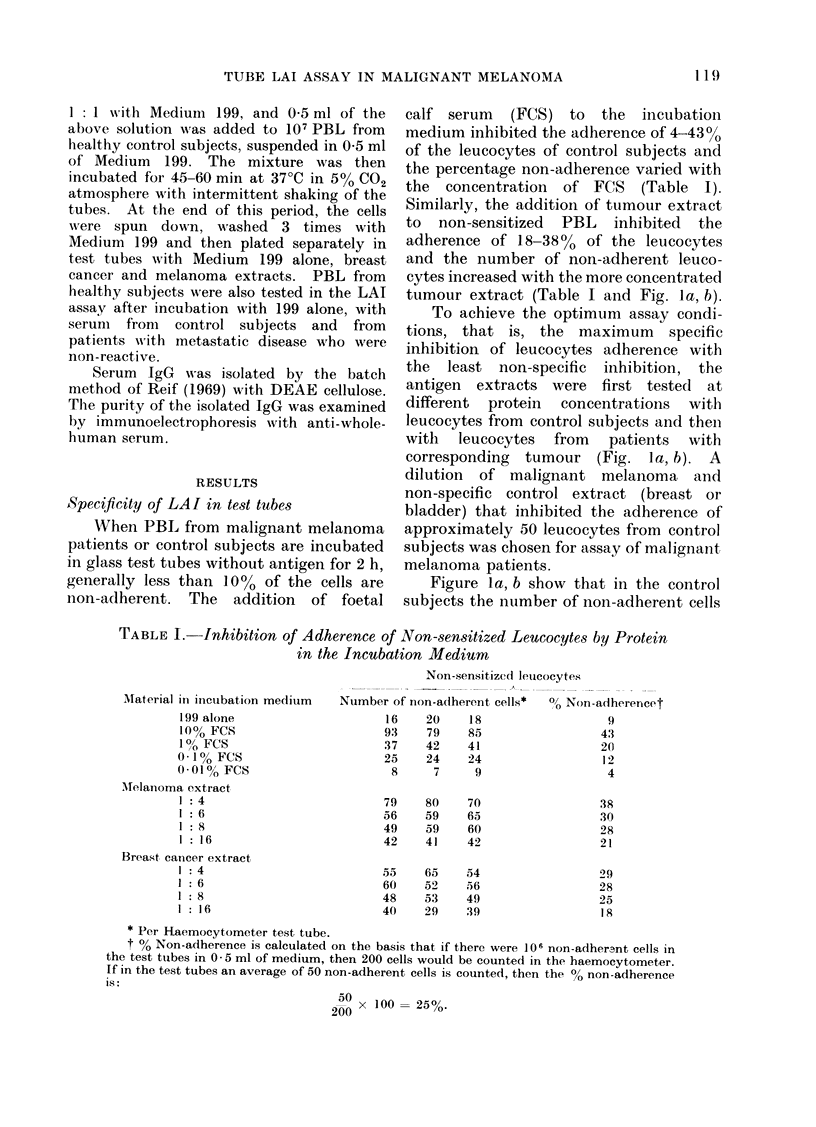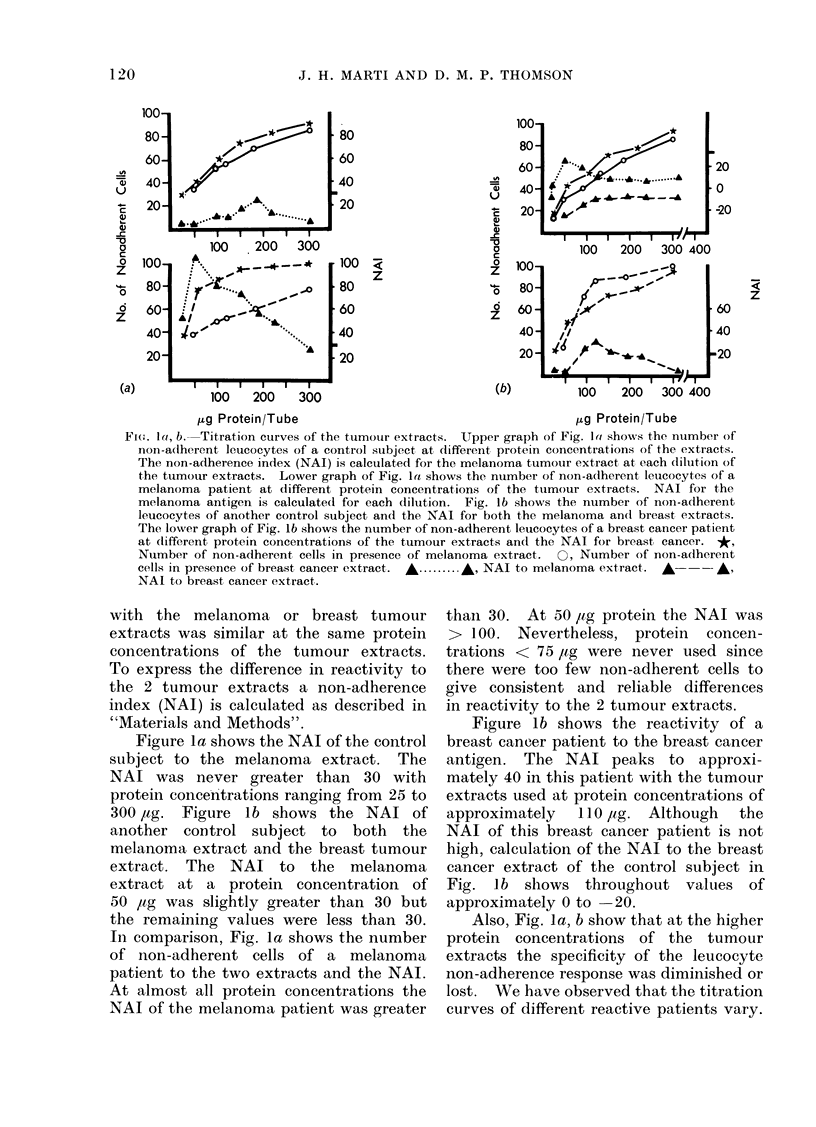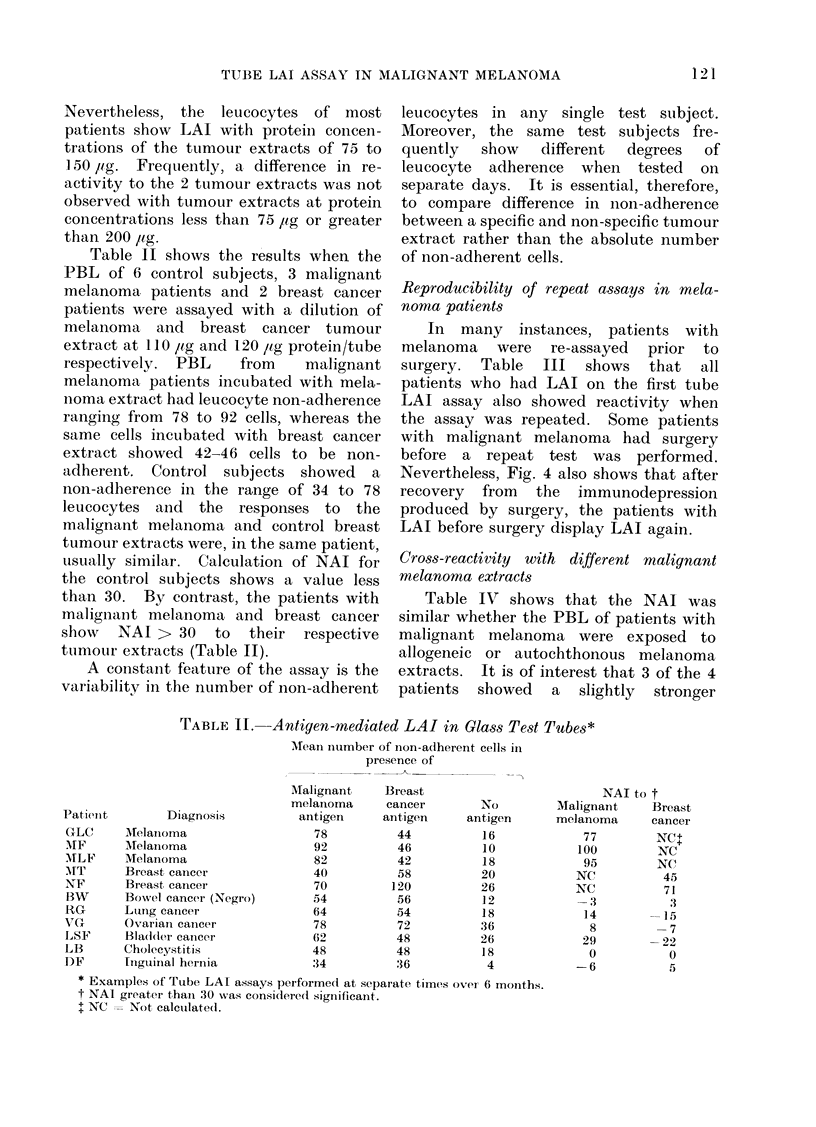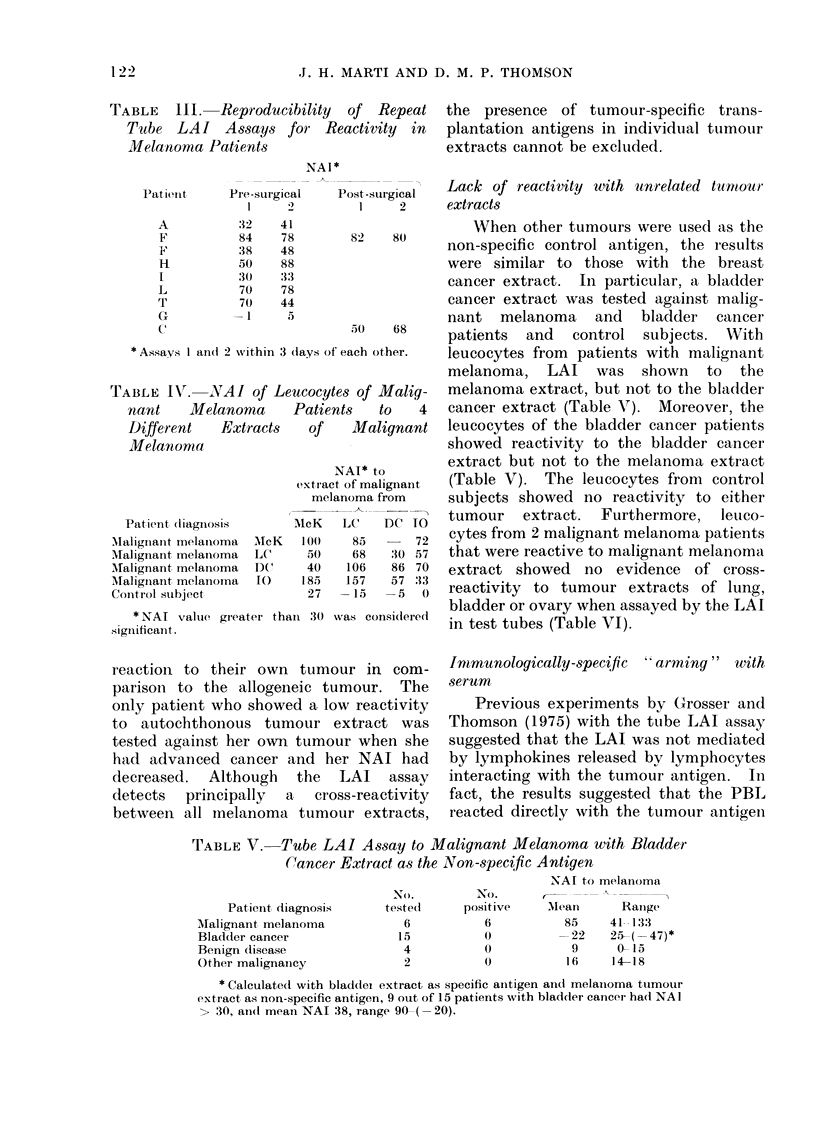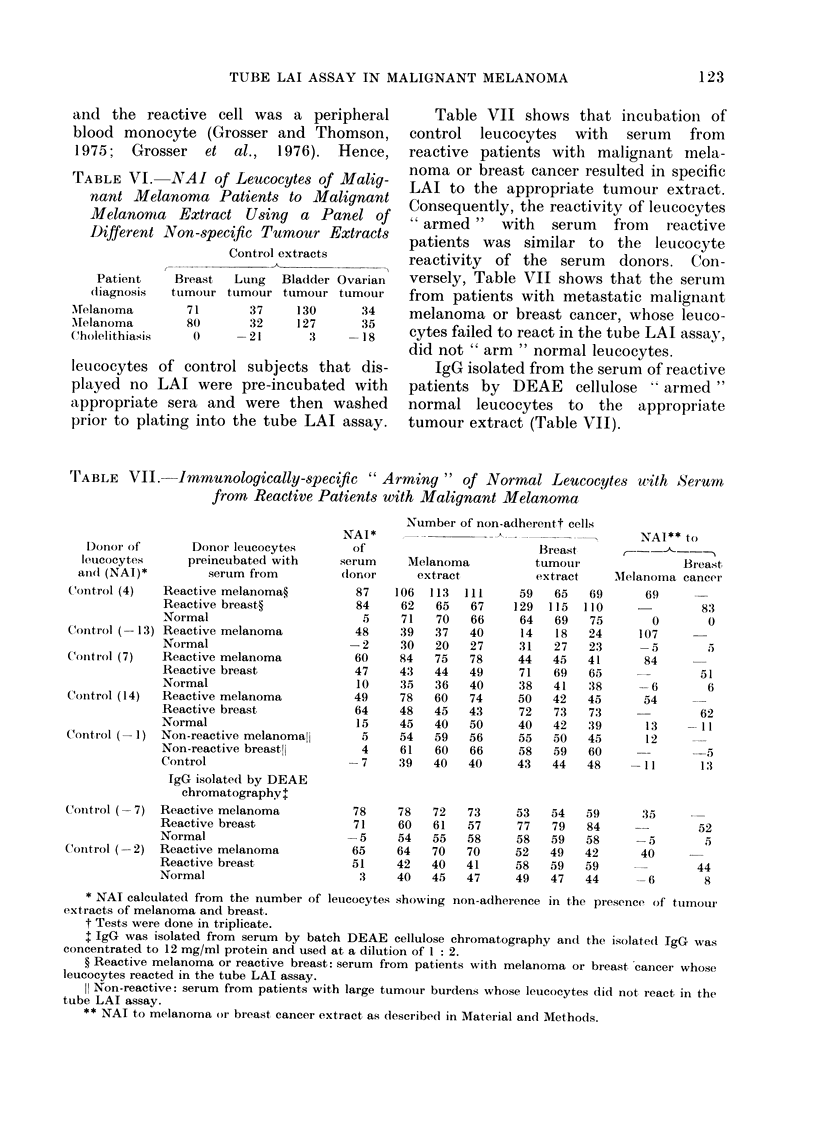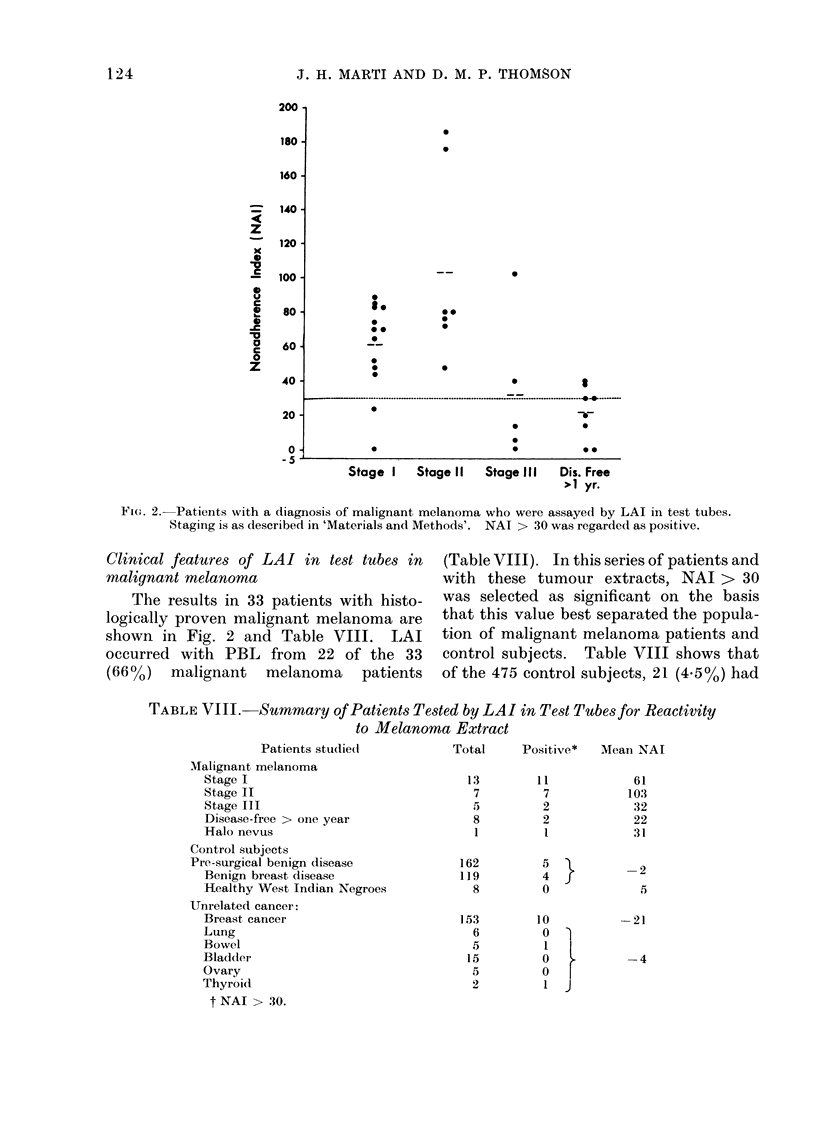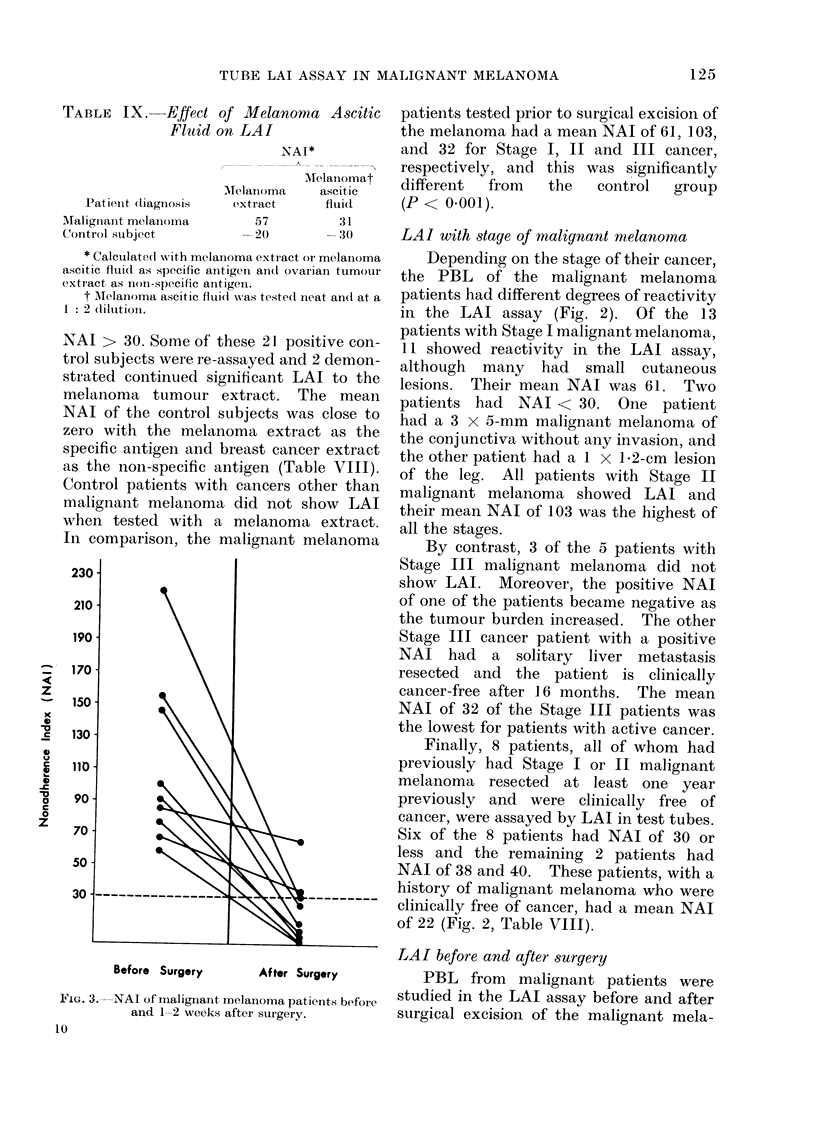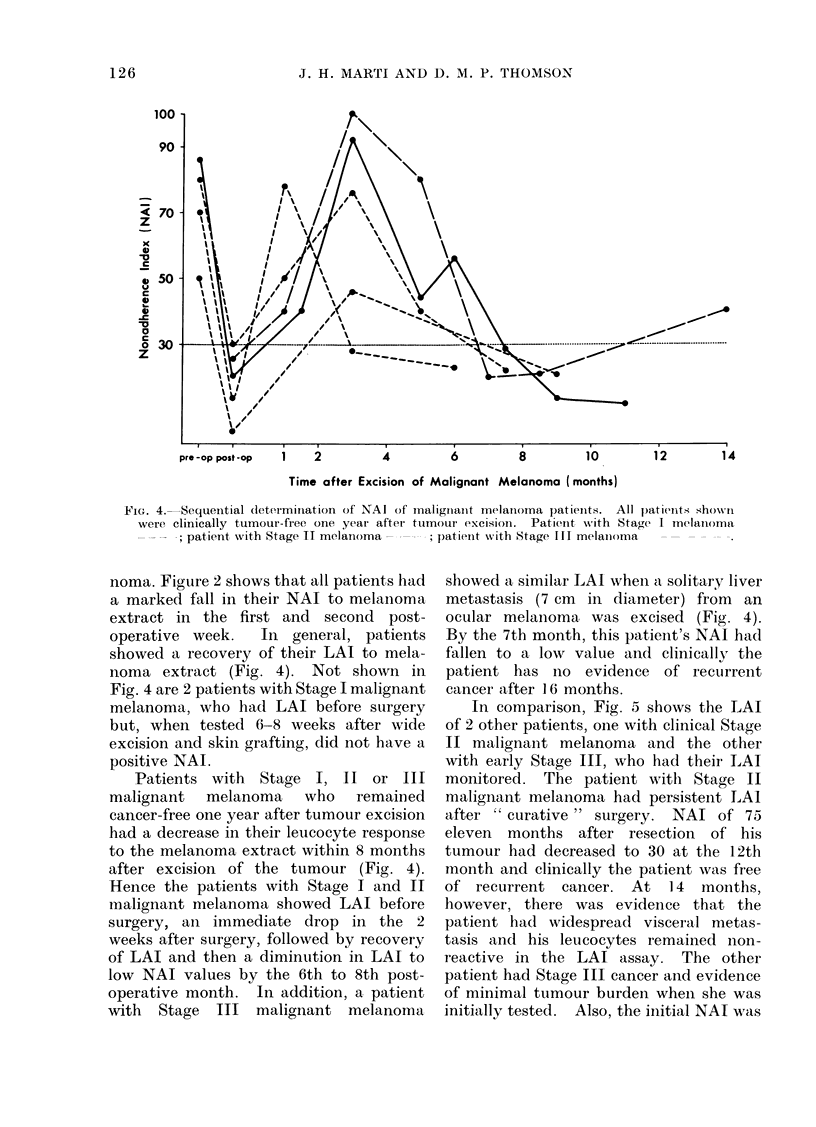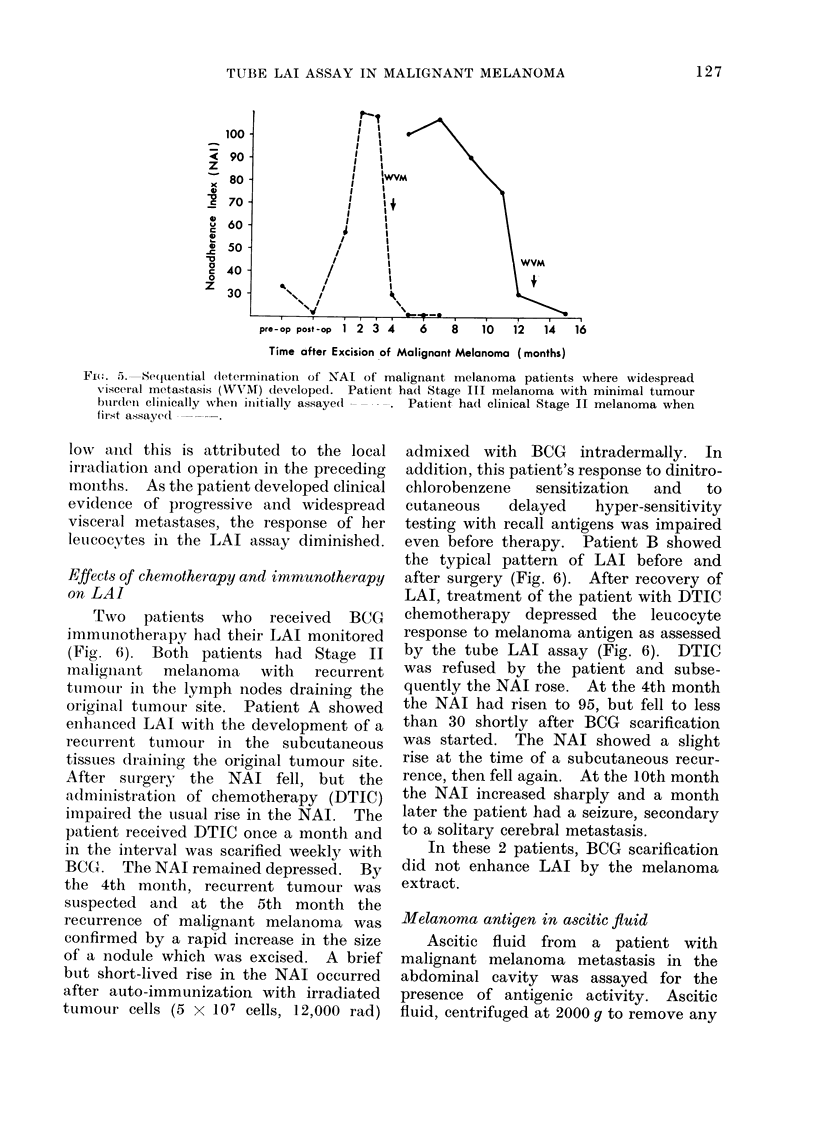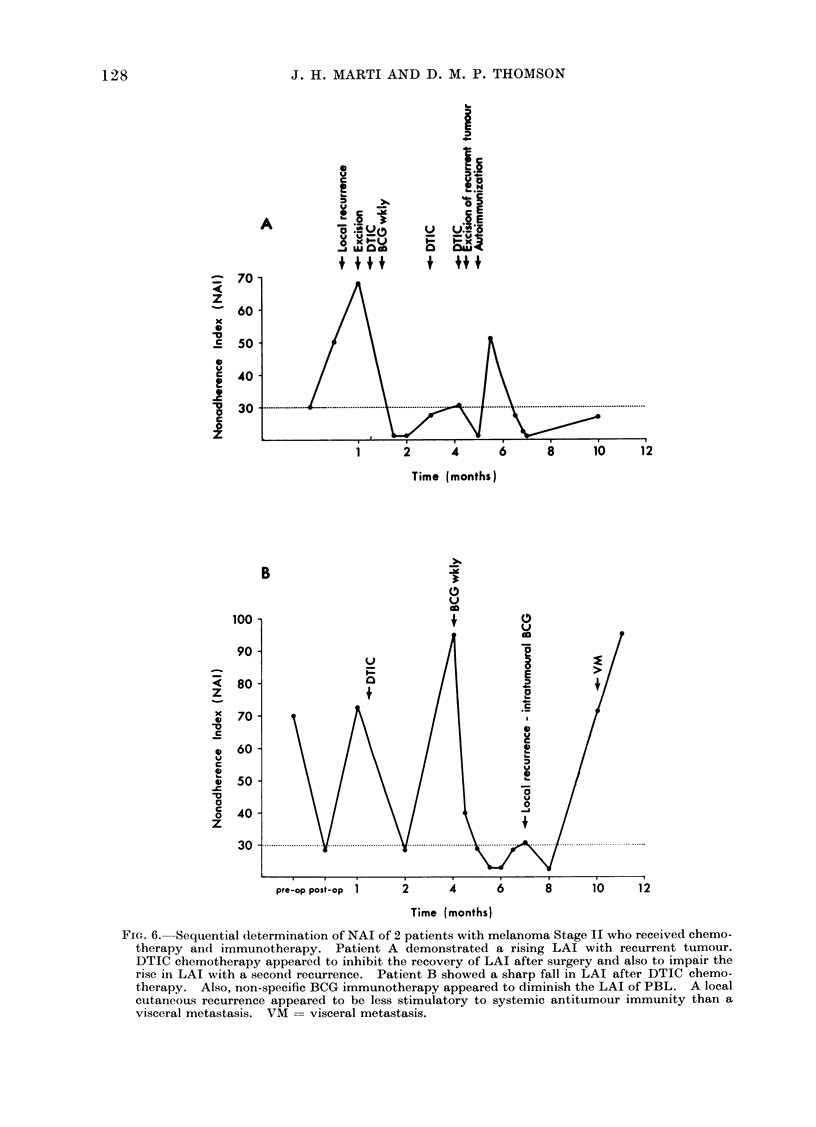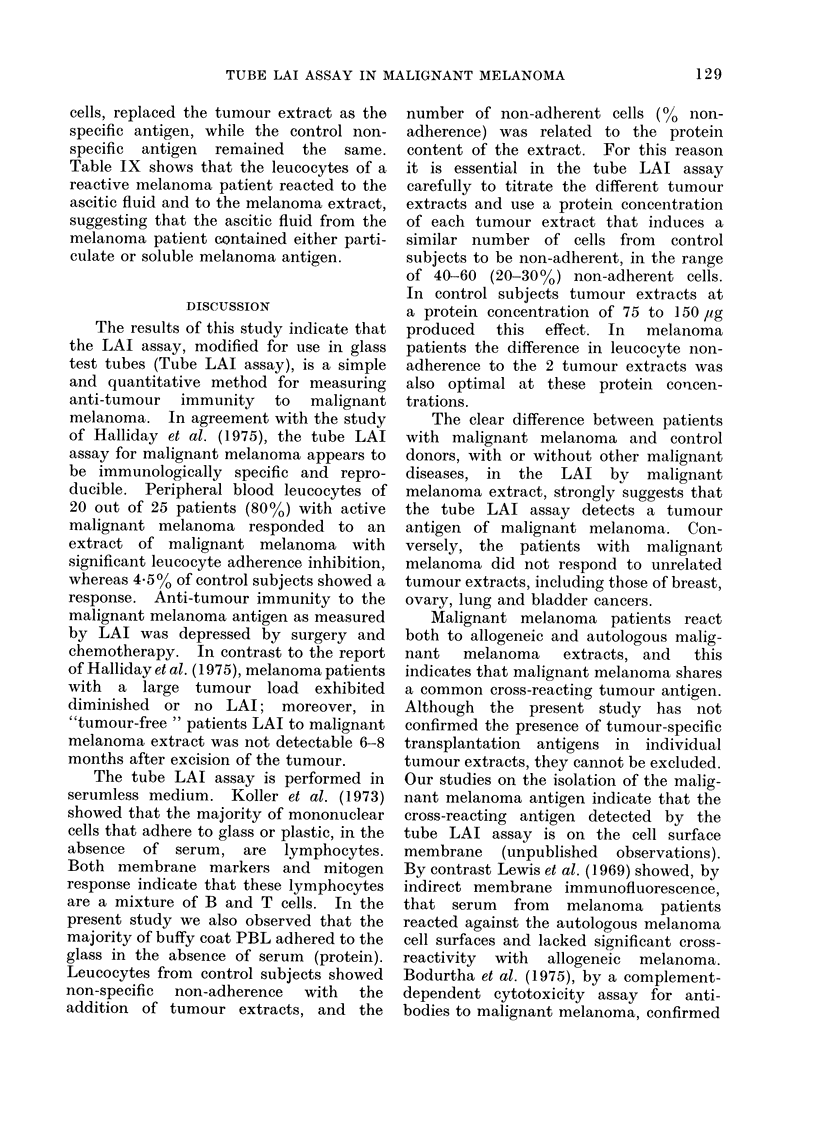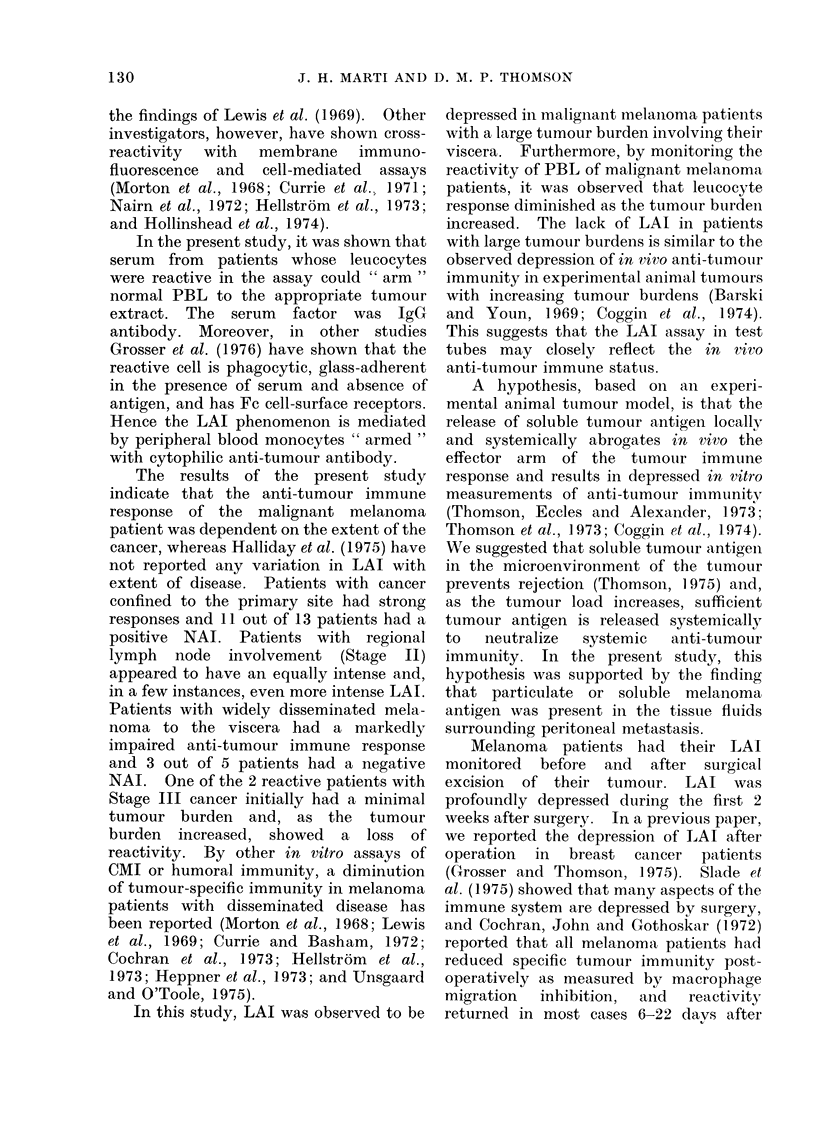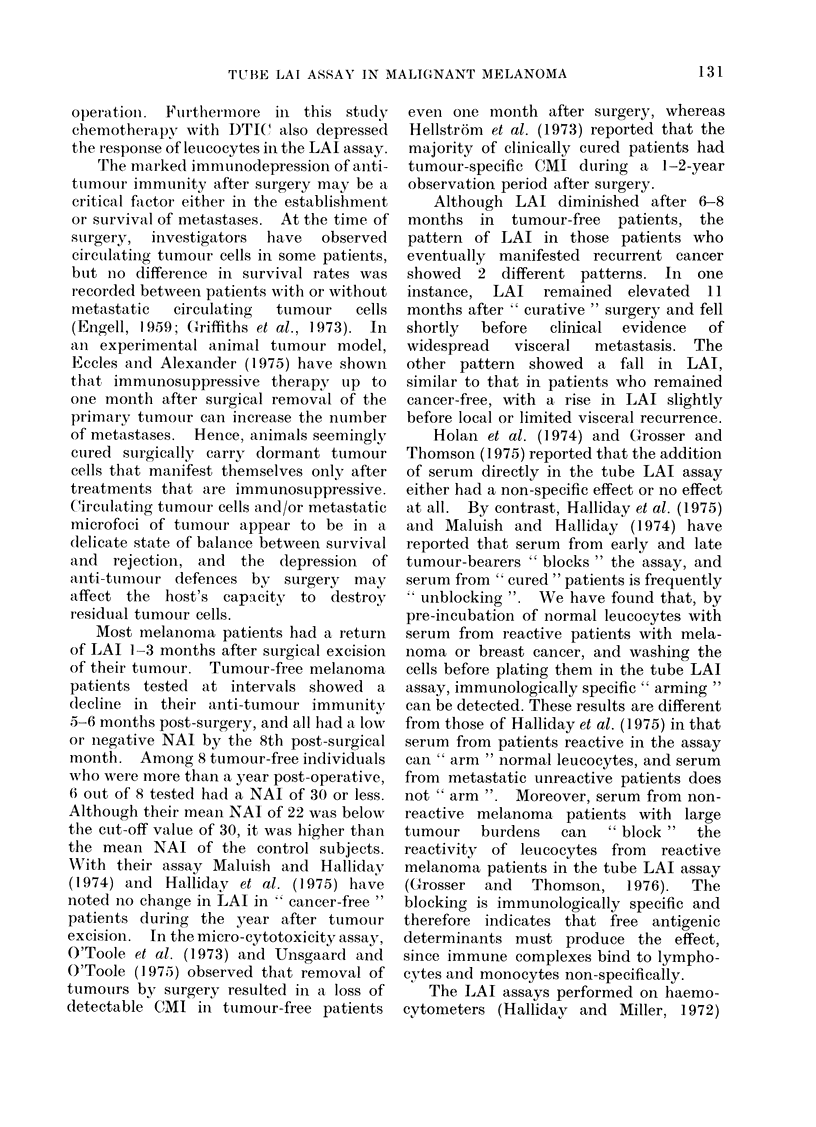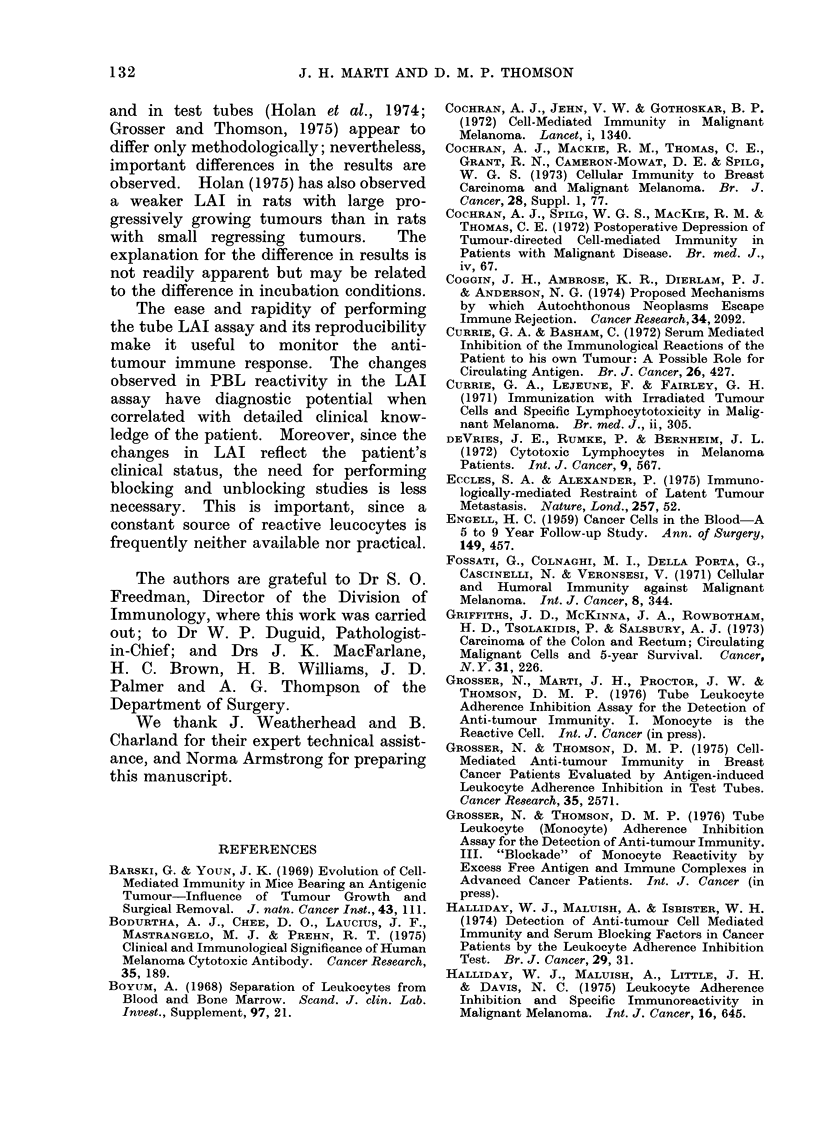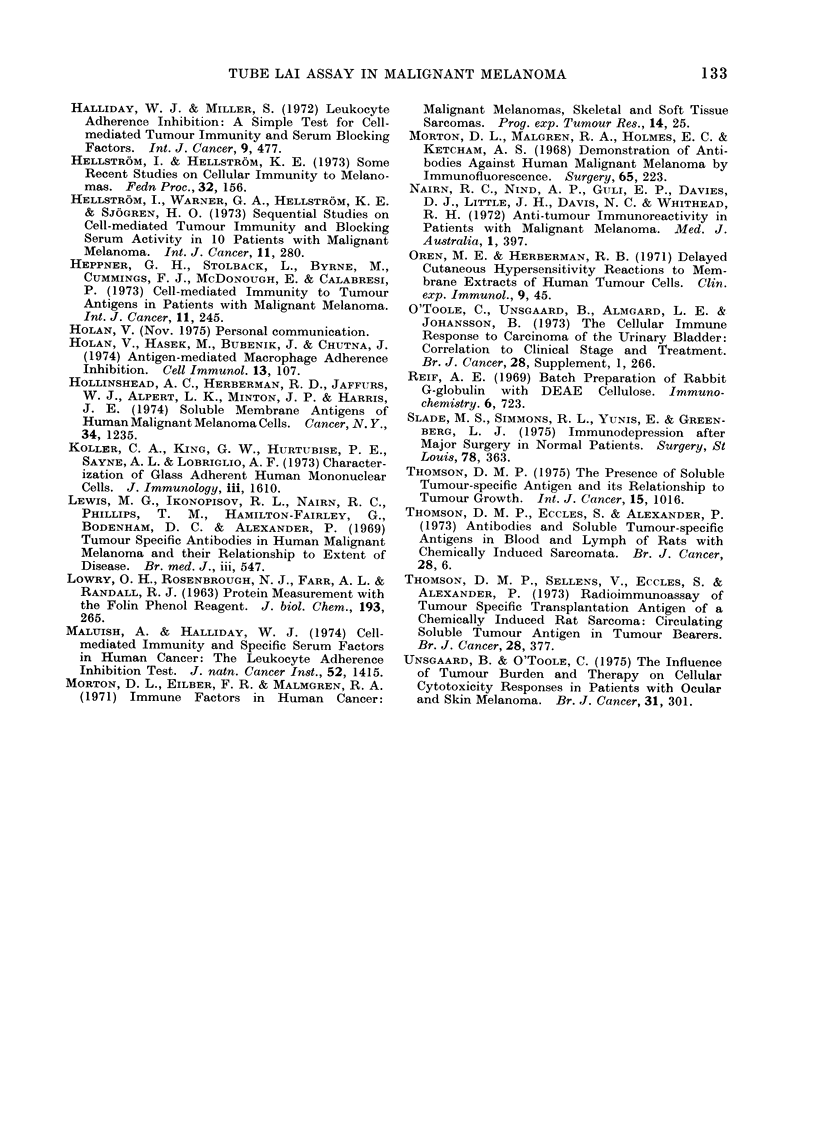# Anti-tumour immunity in malignant melanoma assay by tube leucocyte adherence inhibition.

**DOI:** 10.1038/bjc.1976.134

**Published:** 1976-08

**Authors:** J. H. Marti, D. M. Thomson

## Abstract

Tumour antigen-induced inhibition of leucocyte adherence was modified for use in glass test tubes (Tube LAI assay) for the study of cell-mediated anti-tumour immunity to human malignant melanoma. Peripheral blood leucocytes (PBL) of 20 out of 25 patients (80%) with active malignant melanoma responded to an extract of malignant melanoma with LAI, whereas only 4-5% of 475 control subjects showed a response. The malignant melanoma patients reacted to both allogeneic and autologous extracts of malignant melanoma which indicates a common cross-reacting antigen. Malignant melanoma patients did not respond to unrelated tumour extracts. The LAI was mediated by PBL (monocytes) "armed" with cytophilic anti-tumour antibody specific for the sensitizing tumour antigen. The anti-tumour response of the malignant melanoma patients was dependent on the stage of the cancer, and 11 out of 13 Stage I patients had a positive NAI, whereas patients with disseminated cancer had decreased response. The diminished LAI in patients with large tumour burdens appeared to be the result of release of tumour antigen systemically. Also, surgery and chemotherapy depressed LAI. Although LAI was depressed after surgical excision of the cutaneous melanoma, most patients showed LAI 1-3 months later. Tumour-free melanoma patients monitored for one year by the Tube LAI assay showed a decline in their anti-tumour immunity 5-6 months after surgery. The NAI was low or negative after the 8th post-surgical month in tumour-free patients. Patients with residual malignant melanoma showed persistent or recurrent LAI after the 8th post-surgical month. LAI reactivity monitored after "curative" surgery for malignant melanoma may assist in determining whether the patient is tumour-free or has a recurrence.


*Br. J. Cancer* (1976) **34**, 116

### ANTI-TUMOUR IMMUNITY IN MALIGNANT MELANOMA ASSAY BY TUBE LEUCOCYTE ADHERENCE INHIBITION*

J. H. MARTI† AND D. M. P. THOMSON‡§

*From the Montreal General Hospital Research Institute, Division of Clinical Immunology, Department of Medicine, McGill University, Montreal, Quebec*

Received 19 January 1976 Accepted 20 April 1976

**Summary.—Tumour antigen-induced inhibition of leucocyte adherence was modified for use in glass test tubes (Tube LAI assay) for the study of cell-mediated anti-tumour immunity to human malignant melanoma. Peripheral blood leucocytes (PBL) of 20 out of 25 patients (80%) with active malignant melanoma responded to an extract of malignant melanoma with LAI, whereas only 4·5% of 475 control subjects showed a response. The malignant melanoma patients reacted to both allogeneic and autologous extracts of malignant melanoma which indicates a common cross-reacting antigen. Malignant melanoma patients did not respond to unrelated tumour extracts. The LAI was mediated by PBL (monocytes) " armed " with cytophilic anti-tumour antibody specific for the sensitizing tumour antigen.**

**The anti-tumour response of the malignant melanoma patients was dependent on the stage of the cancer, and 11 out of 13 Stage I patients had a positive NAI, whereas patients with disseminated cancer had decreased response. The diminished LAI in patients with large tumour burdens appeared to be the result of release of tumour antigen systemically. Also, surgery and chemotherapy depressed LAI. Although LAI was depressed after surgical excision of the cutaneous melanoma, most patients showed LAI 1–3 months later. Tumour-free melanoma patients monitored for one year by the Tube LAI assay showed a decline in their anti-tumour immunity 5–6 months after surgery. The NAI was low or negative after the 8th post-surgical month in tumour-free patients. Patients with residual malignant melanoma showed persistent or recurrent LAI after the 8th post-surgical month. LAI reactivity monitored after " curative " surgery for malignant melanoma may assist in determining whether the patient is tumour-free or has a recurrence.**

*Abbreviations used are:*

CMI, Cell-mediated immunity;
DTIC, 5-(3, 3-Dimethyl-1-triazenoimidazole-4-carboxamide;
LAI, Leucocyte adherence inhibition;
NAI, Non-adherence index;
PBL, Peripheral blood leucocytes;
PBS, Phosphate-buffered saline.

EVIDENCE for the existence of a host immune response to malignant melanoma has increased during the last few years (Lewis *et al.*, 1969; Oren and Herberman, 1971; Cochran *et al.*, 1972; deVries, Rumke and Bernheim, 1972; Hellström *et al.*, Heppner *et al.*, 1973; and Hollinshead *et al.*, 1974). Distinctive cellular and humoral immune responses are described (Morton *et al.*, 1968; Lewis *et al.*, 1969; Currie *et al.*, 1971; Fossati *et al.*, 1971; Nairn *et al.*, 1972; Hellström and Hellström, 1973; and Bodurtha *et al.*, 1975). Moreover, quantitative changes in the cellular and humoral immune

* This work was supported by the Medical Research Council of Canada.
† Resident in Surgery, The Montreal General Hospital.
‡ Scholar of the Medical Research Council of Canada.
§ To whom correspondence and reprint requests should be addressed.

responses to tumour are observed with tumour progression (Lewis *et al.*, 1969; Morton, Eilber and Malmgren, 1971; deVries *et al.*, 1972; Cochran *et al.*, 1973; Hellström *et al.*, 1973; and Hellström and Hellström, 1973).

Halliday and Miller (1972) developed an assay of cell-mediated anti-tumour immunity, called leucocyte adherence inhibition (LAI). This assay is based on the findings that non-sensitized leucocytes from both patients with cancer and control subjects adhere to glass, whereas leucocytes from cancer patients but not from control subjects, when mixed *in vitro* with antigenic extracts of tumours of the same histologic type, undergo a diminution in their normal adherence to glass surfaces. In a rat model, Holan *et al.* (1974) described a modified version of the LAI assay that was performed in test tubes and the number of non-adherent cells was measured. We modified the LAI procedure of Holan *et al.*, and studied the anti-tumour immunity to human breast cancer (Grosser and Thomson, 1975).

Recently Halliday *et al.* (1975) reported the results of the LAI assay in 75 patients with malignant melanoma. We studied LAI in 33 patients with malignant melanoma by the LAI assay in test tubes (Tube LAI assay), an assay that appears to differ only methodologically. Nevertheless, there are important differences in the results in the two studies. In the present study we show that LAI depends on the extent of the malignant melanoma, and patients with large tumour loads were unreactive. In addition, surgery and chemotherapy altered LAI. Furthermore, when the kinetics of LAI were monitored during the year after excision of the primary melanoma, LAI reactivity was generally undetectable 6–8 months post-surgery in "cancer-free" patients.

### MATERIALS AND METHODS

*Subject studies.*—Heparinized blood samples were obtained by venepuncture from 33 patients with malignant melanoma. Thirty patients had cutaneous melanoma and 3 patients had ocular melanoma (one primary and 2 secondary). The group of 475 control subjects was composed of 119 patients with benign breast disease, 153 patients with adenocarcinoma of the breast, 162 elective surgical patients with benign disease and 33 patients with other cancers. In addition, 8 healthy West Indian negroes were tested.

Malignant melanoma of the skin was staged as follows: Stage I—a localized primary malignant melanoma confined to the skin or eye; Stage II—regional lymph node metastasis or local recurrence of tumour in skin or lymph nodes; Stage III—distant metastasis.

In some instances, patients with Stage I melanoma were assayed after punch biopsy of the suspect skin lesion, but always before definitive wide surgical excision and skin grafting. Patients with Stage II melanoma were assayed prior to complete surgical excision of the malignant melanoma. Patients with Stage III melanoma had previously had their lesion excised and were presenting with recurrent tumour when they were assayed. In addition, there were 8 patients who had had a cutaneous malignant melanoma (Stage I or II) excised at least a year earlier and they were clinically cancer-free when they were tested for the first time.

All subjects who gave blood samples had the nature of the test explained to them and verbal consent was obtained.

*Tumour extracts.*—Malignant melanoma samples were received at operation and placed in a sterile container. Appropriate amounts of the tumours were taken for routine pathology. Fatty and fibrous tissues were dissected away, and the specimen was finely minced with sharp scissors in ice-cold PBS (0·01 M phosphate buffer, 0·15 M saline) at pH 7·3. The resulting material was homogenized for 10–15 min in 5 volumes of PBS at 40,000 rev/min in a Virtris 45 homogenizer. The homogenate was centrifuged at 20,000 *g* for 30 min and the supernatants were stored at −40°C in 2-ml aliquots. Extracts of other tumours were prepared in an identical fashion. The protein concentrations of the stock extracts of malignant melanoma, breast cancer, and other tumours were 6–8·5 mg/ml. All tumour extracts were of similar protein concentrations. LAI assay in test tubes was performed with a stock extract composed of a pool of 3 different melanoma tumour extracts, whereas individual breast or bladder tumour samples were employed as the non-specific control extracts.

For use in the assay, the concentrated stock extracts were thawed in a water bath at 20°C and a sample was diluted with Medium 199 (Gibco, Grand Island, N.Y.). An 0·1-ml aliquot of the diluted stock extract was added to certain designated tubes containing the standard quantity of $10^6$ PBL in 0·1 ml of Medium 199.

*Antigen-induced leucocyte adherence inhibition (LAI) to glass in test tubes (Tube LAI assay).*—The LAI assay in test tubes was performed as previously described by Grosser and Thomson (1975). In brief, 20-ml samples of heparinized venous blood were obtained in two 10-ml green stopper vacutainer tubes (Becton, Dickinson & Co., Mississauga, Ont.) and incubated vertically at 37°C for 1 h. The resulting leucocyte-rich plasma fraction was aspirated and centrifuged at 200 *g* for 5 min. The cell-free plasma was then removed and discarded. The cell button was suspended in an ice-cold, isotonic, Tris-buffered $NH_4Cl$ solution (Boyum, 1968) by repeated pipetting, and was left for 15 min at 4°C in order to lyse contaminating erythrocytes. This procedure was terminated by the addition of 3 ml of Medium 199.

The cells were centrifuged as described above, and the supernatant was removed and discarded. The cells were then washed twice with 10 ml of Medium 199, and resuspended at a concentration of $10^7$ cells/ml of Medium 199.

Antigen-induced inhibition of leucocyte adherence to glass was tested in 20-ml, 16 × 150 mm test tubes (Kimax, Fisher Scientific, Montreal, Canada). Aliquots of 0·1 ml of a PBL suspension ($10^7$/ml) were placed in the test tubes. Then either 0·1 ml of Medium 199 or 0·1 ml of specific tumour extract or unrelated tumour extract was added to each tube. The mixture was brought to a final volume of 0·5 ml in all tubes by the addition of the appropriate volume of Medium 199. The suspension in each tube was thoroughly mixed with a Pasteur pipette and the tubes were then incubated horizontally, so that the contents covered $\frac{3}{4}$ of the lower surface of each tube. The tubes were incubated at 37°C in a humidified atmosphere of 5% $CO_2$ in air. After 2 h of incubation, the tubes were placed vertically and their contents were agitated with a Pasteur pipette just before counting. The number of non-adherent cells/ml was counted in an improved Nebauer haemocytometer. All assays were in triplicate. Incubation of the cells in the absence of tumour extract beyond a 2-h period did not produce appreciably increased adherence of leucocytes to glass. Hence a 2-h incubation period was chosen.

The results were expressed as:

$$\text{Non-adherence index (NAI)} = \frac{\text{Non-adherent cells in presence of specific antigen} - \text{Non-adherent cells in presence of non-specific antigen}}{\text{Non-adherent cells in presence of non-specific antigen}} \times 100$$

Since this aspect of the study involved delineating the reactivity of patients to malignant melanoma, the specific antigen was an extract of malignant melanoma, while the unrelated antigens were extracts of breast cancer and bladder cancer. Extracts of other tumours, such as cancers of ovary, bladder and lung, were prepared in a similar manner and also employed as the non-specific control antigen or for the study of the specificity of LAI.

*Titration of tumour extracts.*—The stock tumour extracts were diluted and added to the test tubes at different protein concentrations. Figure 1 shows that protein concentrations of approximately 110 $\mu$g and 120 $\mu$g for malignant melanoma and breast tumour extracts respectively appear to be optimal. The tumour extracts were used throughout this study at these dilutions. In this study, other tumour extracts were used at approximately 100 $\mu$g protein/test tube. Protein determinations were performed by the method of Lowry *et al.* (1963) using bovine serum albumin as a standard.

*"Arming" of control cells by serum from reactive melanoma patients.*—Blood was taken from melanoma and breast cancer patients and control subjects and immediately stored at 4°C. After overnight retraction of the clot, the serum was separated and stored at −40°C.

Patients with Stage I or Stage II malignant melanoma and breast cancer had their serum assayed for "arming" in the LAI assay. Before use, the serum was diluted

1 : 1 with Medium 199, and 0·5 ml of the above solution was added to $10^7$ PBL from healthy control subjects, suspended in 0·5 ml of Medium 199. The mixture was then incubated for 45–60 min at 37°C in 5% $CO_2$ atmosphere with intermittent shaking of the tubes. At the end of this period, the cells were spun down, washed 3 times with Medium 199 and then plated separately in test tubes with Medium 199 alone, breast cancer and melanoma extracts. PBL from healthy subjects were also tested in the LAI assay after incubation with 199 alone, with serum from control subjects and from patients with metastatic disease who were non-reactive.

Serum IgG was isolated by the batch method of Reif (1969) with DEAE cellulose. The purity of the isolated IgG was examined by immunoelectrophoresis with anti-whole-human serum.

### RESULTS

### *Specificity of LAI in test tubes*

When PBL from malignant melanoma patients or control subjects are incubated in glass test tubes without antigen for 2 h, generally less than 10% of the cells are non-adherent. The addition of foetal calf serum (FCS) to the incubation medium inhibited the adherence of 4–43% of the leucocytes of control subjects and the percentage non-adherence varied with the concentration of FCS (Table I). Similarly, the addition of tumour extract to non-sensitized PBL inhibited the adherence of 18–38% of the leucocytes and the number of non-adherent leucocytes increased with the more concentrated tumour extract (Table I and Fig. 1*a*, *b*).

To achieve the optimum assay conditions, that is, the maximum specific inhibition of leucocytes adherence with the least non-specific inhibition, the antigen extracts were first tested at different protein concentrations with leucocytes from control subjects and then with leucocytes from patients with corresponding tumour (Fig. 1*a*, *b*). A dilution of malignant melanoma and non-specific control extract (breast or bladder) that inhibited the adherence of approximately 50 leucocytes from control subjects was chosen for assay of malignant melanoma patients.

Figure 1*a*, *b* show that in the control subjects the number of non-adherent cells

TABLE I.—*Inhibition of Adherence of Non-sensitized Leucocytes by Protein in the Incubation Medium*

| Material in incubation medium | Non-sensitized leucocytes: Number of non-adherent cells* | | | % Non-adherence† |
|---|---|---|---|---|
| 199 alone | 16 | 20 | 18 | 9 |
| 10% FCS | 93 | 79 | 85 | 43 |
| 1% FCS | 37 | 42 | 41 | 20 |
| 0·1% FCS | 25 | 24 | 24 | 12 |
| 0·01% FCS | 8 | 7 | 9 | 4 |
| Melanoma extract | | | | |
| 1 : 4 | 79 | 80 | 70 | 38 |
| 1 : 6 | 56 | 59 | 65 | 30 |
| 1 : 8 | 49 | 59 | 60 | 28 |
| 1 : 16 | 42 | 41 | 42 | 21 |
| Breast cancer extract | | | | |
| 1 : 4 | 55 | 65 | 54 | 29 |
| 1 : 6 | 60 | 52 | 56 | 28 |
| 1 : 8 | 48 | 53 | 49 | 25 |
| 1 : 16 | 40 | 29 | 39 | 18 |

* Per Haemocytometer test tube.

† % Non-adherence is calculated on the basis that if there were $10^6$ non-adherent cells in the test tubes in 0·5 ml of medium, then 200 cells would be counted in the haemocytometer. If in the test tubes an average of 50 non-adherent cells is counted, then the % non-adherence is:

$$\frac{50}{200} \times 100 = 25\%.$$

FIG. 1*a*, *b*.—Titration curves of the tumour extracts. Upper graph of Fig. 1*a* shows the number of non-adherent leucocytes of a control subject at different protein concentrations of the extracts. The non-adherence index (NAI) is calculated for the melanoma tumour extract at each dilution of the tumour extracts. Lower graph of Fig. 1*a* shows the number of non-adherent leucocytes of a melanoma patient at different protein concentrations of the tumour extracts. NAI for the melanoma antigen is calculated for each dilution. Fig. 1*b* shows the number of non-adherent leucocytes of another control subject and the NAI for both the melanoma and breast extracts. The lower graph of Fig. 1*b* shows the number of non-adherent leucocytes of a breast cancer patient at different protein concentrations of the tumour extracts and the NAI for breast cancer. ★, Number of non-adherent cells in presence of melanoma extract. ○, Number of non-adherent cells in presence of breast cancer extract. ▲.........▲, NAI to melanoma extract. ▲— — —▲, NAI to breast cancer extract.

with the melanoma or breast tumour extracts was similar at the same protein concentrations of the tumour extracts. To express the difference in reactivity to the 2 tumour extracts a non-adherence index (NAI) is calculated as described in "Materials and Methods".

Figure 1*a* shows the NAI of the control subject to the melanoma extract. The NAI was never greater than 30 with protein concentrations ranging from 25 to 300 $\mu$g. Figure 1*b* shows the NAI of another control subject to both the melanoma extract and the breast tumour extract. The NAI to the melanoma extract at a protein concentration of 50 $\mu$g was slightly greater than 30 but the remaining values were less than 30. In comparison, Fig. 1*a* shows the number of non-adherent cells of a melanoma patient to the two extracts and the NAI. At almost all protein concentrations the NAI of the melanoma patient was greater than 30. At 50 $\mu$g protein the NAI was $> 100$. Nevertheless, protein concentrations $< 75$ $\mu$g were never used since there were too few non-adherent cells to give consistent and reliable differences in reactivity to the 2 tumour extracts.

Figure 1*b* shows the reactivity of a breast cancer patient to the breast cancer antigen. The NAI peaks to approximately 40 in this patient with the tumour extracts used at protein concentrations of approximately 110 $\mu$g. Although the NAI of this breast cancer patient is not high, calculation of the NAI to the breast cancer extract of the control subject in Fig. 1*b* shows throughout values of approximately 0 to $-20$.

Also, Fig. 1*a*, *b* show that at the higher protein concentrations of the tumour extracts the specificity of the leucocyte non-adherence response was diminished or lost. We have observed that the titration curves of different reactive patients vary.

Nevertheless, the leucocytes of most patients show LAI with protein concentrations of the tumour extracts of 75 to 150 $\mu$g. Frequently, a difference in reactivity to the 2 tumour extracts was not observed with tumour extracts at protein concentrations less than 75 $\mu$g or greater than 200 $\mu$g.

Table II shows the results when the PBL of 6 control subjects, 3 malignant melanoma patients and 2 breast cancer patients were assayed with a dilution of melanoma and breast cancer tumour extract at 110 $\mu$g and 120 $\mu$g protein/tube respectively. PBL from malignant melanoma patients incubated with melanoma extract had leucocyte non-adherence ranging from 78 to 92 cells, whereas the same cells incubated with breast cancer extract showed 42–46 cells to be non-adherent. Control subjects showed a non-adherence in the range of 34 to 78 leucocytes and the responses to the malignant melanoma and control breast tumour extracts were, in the same patient, usually similar. Calculation of NAI for the control subjects shows a value less than 30. By contrast, the patients with malignant melanoma and breast cancer show NAI $> 30$ to their respective tumour extracts (Table II).

A constant feature of the assay is the variability in the number of non-adherent leucocytes in any single test subject. Moreover, the same test subjects frequently show different degrees of leucocyte adherence when tested on separate days. It is essential, therefore, to compare difference in non-adherence between a specific and non-specific tumour extract rather than the absolute number of non-adherent cells.

*Reproducibility of repeat assays in melanoma patients*

In many instances, patients with melanoma were re-assayed prior to surgery. Table III shows that all patients who had LAI on the first tube LAI assay also showed reactivity when the assay was repeated. Some patients with malignant melanoma had surgery before a repeat test was performed. Nevertheless, Fig. 4 also shows that after recovery from the immunodepression produced by surgery, the patients with LAI before surgery display LAI again.

*Cross-reactivity with different malignant melanoma extracts*

Table IV shows that the NAI was similar whether the PBL of patients with malignant melanoma were exposed to allogeneic or autochthonous melanoma extracts. It is of interest that 3 of the 4 patients showed a slightly stronger

TABLE II.—*Antigen-mediated LAI in Glass Test Tubes**

| | | Mean number of non-adherent cells in presence of | | | NAI to † | |
|---|---|---|---|---|---|---|
| Patient | Diagnosis | Malignant melanoma antigen | Breast cancer antigen | No antigen | Malignant melanoma | Breast cancer |
| GLC | Melanoma | 78 | 44 | 16 | 77 | NC‡ |
| MF | Melanoma | 92 | 46 | 10 | 100 | NC |
| MLF | Melanoma | 82 | 42 | 18 | 95 | NC |
| MT | Breast cancer | 40 | 58 | 20 | NC | 45 |
| NF | Breast cancer | 70 | 120 | 26 | NC | 71 |
| BW | Bowel cancer (Negro) | 54 | 56 | 12 | −3 | 3 |
| RG | Lung cancer | 64 | 54 | 18 | 14 | −15 |
| VG | Ovarian cancer | 78 | 72 | 36 | 8 | −7 |
| LSF | Bladder cancer | 62 | 48 | 26 | 29 | −22 |
| LB | Cholecystitis | 48 | 48 | 18 | 0 | 0 |
| DF | Inguinal hernia | 34 | 36 | 4 | −6 | 5 |

* Examples of Tube LAI assays performed at separate times over 6 months.

† NAI greater than 30 was considered significant.

‡ NC = Not calculated.

TABLE III.—*Reproducibility of Repeat Tube LAI Assays for Reactivity in Melanoma Patients*

| Patient | NAI* Pre-surgical 1 | 2 | Post-surgical 1 | 2 |
|---|---|---|---|---|
| A | 32 | 41 | | |
| F | 84 | 78 | 82 | 80 |
| F | 38 | 48 | | |
| H | 50 | 88 | | |
| I | 30 | 33 | | |
| L | 70 | 78 | | |
| T | 70 | 44 | | |
| G | −1 | 5 | | |
| C | | | 50 | 68 |

* Assays 1 and 2 within 3 days of each other.

TABLE IV.—*NAI of Leucocytes of Malignant Melanoma Patients to 4 Different Extracts of Malignant Melanoma*

| Patient diagnosis | | NAI* to extract of malignant melanoma from McK | LC | DC | IO |
|---|---|---|---|---|---|
| Malignant melanoma | McK | 100 | 85 | — | 72 |
| Malignant melanoma | LC | 50 | 68 | 30 | 57 |
| Malignant melanoma | DC | 40 | 106 | 86 | 70 |
| Malignant melanoma | IO | 185 | 157 | 57 | 33 |
| Control subject | | 27 | −15 | −5 | 0 |

* NAI value greater than 30 was considered significant.

reaction to their own tumour in comparison to the allogeneic tumour. The only patient who showed a low reactivity to autochthonous tumour extract was tested against her own tumour when she had advanced cancer and her NAI had decreased. Although the LAI assay detects principally a cross-reactivity between all melanoma tumour extracts, the presence of tumour-specific transplantation antigens in individual tumour extracts cannot be excluded.

*Lack of reactivity with unrelated tumour extracts*

When other tumours were used as the non-specific control antigen, the results were similar to those with the breast cancer extract. In particular, a bladder cancer extract was tested against malignant melanoma and bladder cancer patients and control subjects. With leucocytes from patients with malignant melanoma, LAI was shown to the melanoma extract, but not to the bladder cancer extract (Table V). Moreover, the leucocytes of the bladder cancer patients showed reactivity to the bladder cancer extract but not to the melanoma extract (Table V). The leucocytes from control subjects showed no reactivity to either tumour extract. Furthermore, leucocytes from 2 malignant melanoma patients that were reactive to malignant melanoma extract showed no evidence of cross-reactivity to tumour extracts of lung, bladder or ovary when assayed by the LAI in test tubes (Table VI).

*Immunologically-specific "arming" with serum*

Previous experiments by Grosser and Thomson (1975) with the tube LAI assay suggested that the LAI was not mediated by lymphokines released by lymphocytes interacting with the tumour antigen. In fact, the results suggested that the PBL reacted directly with the tumour antigen

TABLE V.—*Tube LAI Assay to Malignant Melanoma with Bladder Cancer Extract as the Non-specific Antigen*

| Patient diagnosis | No. tested | No. positive | NAI to melanoma Mean | Range |
|---|---|---|---|---|
| Malignant melanoma | 6 | 6 | 85 | 41–133 |
| Bladder cancer | 15 | 0 | −22 | 25–(−47)* |
| Benign disease | 4 | 0 | 9 | 0–15 |
| Other malignancy | 2 | 0 | 16 | 14–18 |

* Calculated with bladder extract as specific antigen and melanoma tumour extract as non-specific antigen, 9 out of 15 patients with bladder cancer had NAI > 30, and mean NAI 38, range 90–(−20).

and the reactive cell was a peripheral blood monocyte (Grosser and Thomson, 1975; Grosser *et al.*, 1976). Hence,

TABLE VI.—*NAI of Leucocytes of Malignant Melanoma Patients to Malignant Melanoma Extract Using a Panel of Different Non-specific Tumour Extracts*

| Patient diagnosis | Control extracts: Breast tumour | Lung tumour | Bladder tumour | Ovarian tumour |
|---|---|---|---|---|
| Melanoma | 71 | 37 | 130 | 34 |
| Melanoma | 80 | 32 | 127 | 35 |
| Cholelithiasis | 0 | −21 | 3 | −18 |

leucocytes of control subjects that displayed no LAI were pre-incubated with appropriate sera and were then washed prior to plating into the tube LAI assay.

Table VII shows that incubation of control leucocytes with serum from reactive patients with malignant melanoma or breast cancer resulted in specific LAI to the appropriate tumour extract. Consequently, the reactivity of leucocytes "armed" with serum from reactive patients was similar to the leucocyte reactivity of the serum donors. Conversely, Table VII shows that the serum from patients with metastatic malignant melanoma or breast cancer, whose leucocytes failed to react in the tube LAI assay, did not "arm" normal leucocytes.

IgG isolated from the serum of reactive patients by DEAE cellulose "armed" normal leucocytes to the appropriate tumour extract (Table VII).

TABLE VII.—*Immunologically-specific "Arming" of Normal Leucocytes with Serum from Reactive Patients with Malignant Melanoma*

| Donor of leucocytes and (NAI)* | Donor leucocytes preincubated with serum from | NAI* of serum donor | Number of non-adherent† cells: Melanoma extract | | | Breast tumour extract | | | NAI** to: Melanoma | Breast cancer |
|---|---|---|---|---|---|---|---|---|---|---|
| Control (4) | Reactive melanoma§ | 87 | 106 | 113 | 111 | 59 | 65 | 69 | 69 | — |
| | Reactive breast§ | 84 | 62 | 65 | 67 | 129 | 115 | 110 | — | 83 |
| | Normal | 5 | 71 | 70 | 66 | 64 | 69 | 75 | 0 | 0 |
| Control (−13) | Reactive melanoma | 48 | 39 | 37 | 40 | 14 | 18 | 24 | 107 | — |
| | Normal | −2 | 30 | 20 | 27 | 31 | 27 | 23 | −5 | 5 |
| Control (7) | Reactive melanoma | 60 | 84 | 75 | 78 | 44 | 45 | 41 | 84 | — |
| | Reactive breast | 47 | 43 | 44 | 49 | 71 | 69 | 65 | — | 51 |
| | Normal | 10 | 35 | 36 | 40 | 38 | 41 | 38 | −6 | 6 |
| Control (14) | Reactive melanoma | 49 | 78 | 60 | 74 | 50 | 42 | 45 | 54 | — |
| | Reactive breast | 64 | 48 | 45 | 43 | 72 | 73 | 73 | — | 62 |
| | Normal | 15 | 45 | 40 | 50 | 40 | 42 | 39 | 13 | −11 |
| Control (−1) | Non-reactive melanoma‖ | 5 | 54 | 59 | 56 | 55 | 50 | 45 | 12 | — |
| | Non-reactive breast‖ | 4 | 61 | 60 | 66 | 58 | 59 | 60 | — | —5 |
| | Control | −7 | 39 | 40 | 40 | 43 | 44 | 48 | −11 | 13 |
| | IgG isolated by DEAE chromatography‡ | | | | | | | | | |
| Control (−7) | Reactive melanoma | 78 | 78 | 72 | 73 | 53 | 54 | 59 | 35 | — |
| | Reactive breast | 71 | 60 | 61 | 57 | 77 | 79 | 84 | — | 52 |
| | Normal | −5 | 54 | 55 | 58 | 58 | 59 | 58 | −5 | 5 |
| Control (−2) | Reactive melanoma | 65 | 64 | 70 | 70 | 52 | 49 | 42 | 40 | — |
| | Reactive breast | 51 | 42 | 40 | 41 | 58 | 59 | 59 | — | 44 |
| | Normal | 3 | 40 | 45 | 47 | 49 | 47 | 44 | −6 | 8 |

* NAI calculated from the number of leucocytes showing non-adherence in the presence of tumour extracts of melanoma and breast.

† Tests were done in triplicate.

‡ IgG was isolated from serum by batch DEAE cellulose chromatography and the isolated IgG was concentrated to 12 mg/ml protein and used at a dilution of 1 : 2.

§ Reactive melanoma or reactive breast: serum from patients with melanoma or breast cancer whose leucocytes reacted in the tube LAI assay.

‖ Non-reactive: serum from patients with large tumour burdens whose leucocytes did not react in the tube LAI assay.

** NAI to melanoma or breast cancer extract as described in Material and Methods.

FIG. 2.—Patients with a diagnosis of malignant melanoma who were assayed by LAI in test tubes. Staging is as described in 'Materials and Methods'. NAI > 30 was regarded as positive.

*Clinical features of LAI in test tubes in malignant melanoma*

The results in 33 patients with histologically proven malignant melanoma are shown in Fig. 2 and Table VIII. LAI occurred with PBL from 22 of the 33 (66%) malignant melanoma patients (Table VIII). In this series of patients and with these tumour extracts, NAI > 30 was selected as significant on the basis that this value best separated the population of malignant melanoma patients and control subjects. Table VIII shows that of the 475 control subjects, 21 (4·5%) had

TABLE VIII.—*Summary of Patients Tested by LAI in Test Tubes for Reactivity to Melanoma Extract*

| Patients studied | Total | Positive* | Mean NAI |
|---|---|---|---|
| Malignant melanoma | | | |
| Stage I | 13 | 11 | 61 |
| Stage II | 7 | 7 | 103 |
| Stage III | 5 | 2 | 32 |
| Disease-free > one year | 8 | 2 | 22 |
| Halo nevus | 1 | 1 | 31 |
| Control subjects | | | |
| Pre-surgical benign disease | 162 | 5 } | −2 |
| Benign breast disease | 119 | 4 } | |
| Healthy West Indian Negroes | 8 | 0 | 5 |
| Unrelated cancer: | | | |
| Breast cancer | 153 | 10 | −21 |
| Lung | 6 | 0 } | −4 |
| Bowel | 5 | 1 } | |
| Bladder | 15 | 0 } | |
| Ovary | 5 | 0 } | |
| Thyroid | 2 | 1 } | |

† NAI > 30.

TABLE IX.—*Effect of Melanoma Ascitic Fluid on LAI*

| | NAI* | |
|---|---|---|
| Patient diagnosis | Melanoma extract | Melanoma† ascitic fluid |
| Malignant melanoma | 57 | 31 |
| Control subject | −20 | −30 |

* Calculated with melanoma extract or melanoma ascitic fluid as specific antigen and ovarian tumour extract as non-specific antigen.

† Melanoma ascitic fluid was tested neat and at a 1 : 2 dilution.

NAI > 30. Some of these 21 positive control subjects were re-assayed and 2 demonstrated continued significant LAI to the melanoma tumour extract. The mean NAI of the control subjects was close to zero with the melanoma extract as the specific antigen and breast cancer extract as the non-specific antigen (Table VIII). Control patients with cancers other than malignant melanoma did not show LAI when tested with a melanoma extract. In comparison, the malignant melanoma patients tested prior to surgical excision of the melanoma had a mean NAI of 61, 103, and 32 for Stage I, II and III cancer, respectively, and this was significantly different from the control group ($P < 0{\cdot}001$).

FIG. 3.—NAI of malignant melanoma patients before and 1–2 weeks after surgery.

*LAI with stage of malignant melanoma*

Depending on the stage of their cancer, the PBL of the malignant melanoma patients had different degrees of reactivity in the LAI assay (Fig. 2). Of the 13 patients with Stage I malignant melanoma, 11 showed reactivity in the LAI assay, although many had small cutaneous lesions. Their mean NAI was 61. Two patients had NAI < 30. One patient had a 3 × 5-mm malignant melanoma of the conjunctiva without any invasion, and the other patient had a 1 × 1·2-cm lesion of the leg. All patients with Stage II malignant melanoma showed LAI and their mean NAI of 103 was the highest of all the stages.

By contrast, 3 of the 5 patients with Stage III malignant melanoma did not show LAI. Moreover, the positive NAI of one of the patients became negative as the tumour burden increased. The other Stage III cancer patient with a positive NAI had a solitary liver metastasis resected and the patient is clinically cancer-free after 16 months. The mean NAI of 32 of the Stage III patients was the lowest for patients with active cancer.

Finally, 8 patients, all of whom had previously had Stage I or II malignant melanoma resected at least one year previously and were clinically free of cancer, were assayed by LAI in test tubes. Six of the 8 patients had NAI of 30 or less and the remaining 2 patients had NAI of 38 and 40. These patients, with a history of malignant melanoma who were clinically free of cancer, had a mean NAI of 22 (Fig. 2, Table VIII).

*LAI before and after surgery*

PBL from malignant patients were studied in the LAI assay before and after surgical excision of the malignant mela-

FIG. 4.—Sequential determination of NAI of malignant melanoma patients. All patients shown were clinically tumour-free one year after tumour excision. Patient with Stage I melanoma - - - -; patient with Stage II melanoma ———; patient with Stage III melanoma — — — —.

noma. Figure 2 shows that all patients had a marked fall in their NAI to melanoma extract in the first and second post-operative week. In general, patients showed a recovery of their LAI to melanoma extract (Fig. 4). Not shown in Fig. 4 are 2 patients with Stage I malignant melanoma, who had LAI before surgery but, when tested 6–8 weeks after wide excision and skin grafting, did not have a positive NAI.

Patients with Stage I, II or III malignant melanoma who remained cancer-free one year after tumour excision had a decrease in their leucocyte response to the melanoma extract within 8 months after excision of the tumour (Fig. 4). Hence the patients with Stage I and II malignant melanoma showed LAI before surgery, an immediate drop in the 2 weeks after surgery, followed by recovery of LAI and then a diminution in LAI to low NAI values by the 6th to 8th post-operative month. In addition, a patient with Stage III malignant melanoma showed a similar LAI when a solitary liver metastasis (7 cm in diameter) from an ocular melanoma was excised (Fig. 4). By the 7th month, this patient's NAI had fallen to a low value and clinically the patient has no evidence of recurrent cancer after 16 months.

In comparison, Fig. 5 shows the LAI of 2 other patients, one with clinical Stage II malignant melanoma and the other with early Stage III, who had their LAI monitored. The patient with Stage II malignant melanoma had persistent LAI after " curative " surgery. NAI of 75 eleven months after resection of his tumour had decreased to 30 at the 12th month and clinically the patient was free of recurrent cancer. At 14 months, however, there was evidence that the patient had widespread visceral metastasis and his leucocytes remained non-reactive in the LAI assay. The other patient had Stage III cancer and evidence of minimal tumour burden when she was initially tested. Also, the initial NAI was

FIG. 5.—Sequential determination of NAI of malignant melanoma patients where widespread visceral metastasis (WVM) developed. Patient had Stage III melanoma with minimal tumour burden clinically when initially assayed – – · · –. Patient had clinical Stage II melanoma when first assayed ———.

low and this is attributed to the local irradiation and operation in the preceding months. As the patient developed clinical evidence of progressive and widespread visceral metastases, the response of her leucocytes in the LAI assay diminished.

*Effects of chemotherapy and immunotherapy on LAI*

Two patients who received BCG immunotherapy had their LAI monitored (Fig. 6). Both patients had Stage II malignant melanoma with recurrent tumour in the lymph nodes draining the original tumour site. Patient A showed enhanced LAI with the development of a recurrent tumour in the subcutaneous tissues draining the original tumour site. After surgery the NAI fell, but the administration of chemotherapy (DTIC) impaired the usual rise in the NAI. The patient received DTIC once a month and in the interval was scarified weekly with BCG. The NAI remained depressed. By the 4th month, recurrent tumour was suspected and at the 5th month the recurrence of malignant melanoma was confirmed by a rapid increase in the size of a nodule which was excised. A brief but short-lived rise in the NAI occurred after auto-immunization with irradiated tumour cells ($5 \times 10^7$ cells, 12,000 rad) admixed with BCG intradermally. In addition, this patient's response to dinitrochlorobenzene sensitization and to cutaneous delayed hyper-sensitivity testing with recall antigens was impaired even before therapy. Patient B showed the typical pattern of LAI before and after surgery (Fig. 6). After recovery of LAI, treatment of the patient with DTIC chemotherapy depressed the leucocyte response to melanoma antigen as assessed by the tube LAI assay (Fig. 6). DTIC was refused by the patient and subsequently the NAI rose. At the 4th month the NAI had risen to 95, but fell to less than 30 shortly after BCG scarification was started. The NAI showed a slight rise at the time of a subcutaneous recurrence, then fell again. At the 10th month the NAI increased sharply and a month later the patient had a seizure, secondary to a solitary cerebral metastasis.

In these 2 patients, BCG scarification did not enhance LAI by the melanoma extract.

*Melanoma antigen in ascitic fluid*

Ascitic fluid from a patient with malignant melanoma metastasis in the abdominal cavity was assayed for the presence of antigenic activity. Ascitic fluid, centrifuged at 2000 *g* to remove any

FIG. 6.—Sequential determination of NAI of 2 patients with melanoma Stage II who received chemotherapy and immunotherapy. Patient A demonstrated a rising LAI with recurrent tumour. DTIC chemotherapy appeared to inhibit the recovery of LAI after surgery and also to impair the rise in LAI with a second recurrence. Patient B showed a sharp fall in LAI after DTIC chemotherapy. Also, non-specific BCG immunotherapy appeared to diminish the LAI of PBL. A local cutaneous recurrence appeared to be less stimulatory to systemic antitumour immunity than a visceral metastasis. VM = visceral metastasis.

cells, replaced the tumour extract as the specific antigen, while the control non-specific antigen remained the same. Table IX shows that the leucocytes of a reactive melanoma patient reacted to the ascitic fluid and to the melanoma extract, suggesting that the ascitic fluid from the melanoma patient contained either particulate or soluble melanoma antigen.

### DISCUSSION

The results of this study indicate that the LAI assay, modified for use in glass test tubes (Tube LAI assay), is a simple and quantitative method for measuring anti-tumour immunity to malignant melanoma. In agreement with the study of Halliday *et al.* (1975), the tube LAI assay for malignant melanoma appears to be immunologically specific and reproducible. Peripheral blood leucocytes of 20 out of 25 patients (80%) with active malignant melanoma responded to an extract of malignant melanoma with significant leucocyte adherence inhibition, whereas 4·5% of control subjects showed a response. Anti-tumour immunity to the malignant melanoma antigen as measured by LAI was depressed by surgery and chemotherapy. In contrast to the report of Halliday *et al.* (1975), melanoma patients with a large tumour load exhibited diminished or no LAI; moreover, in "tumour-free " patients LAI to malignant melanoma extract was not detectable 6–8 months after excision of the tumour.

The tube LAI assay is performed in serumless medium. Koller *et al.* (1973) showed that the majority of mononuclear cells that adhere to glass or plastic, in the absence of serum, are lymphocytes. Both membrane markers and mitogen response indicate that these lymphocytes are a mixture of B and T cells. In the present study we also observed that the majority of buffy coat PBL adhered to the glass in the absence of serum (protein). Leucocytes from control subjects showed non-specific non-adherence with the addition of tumour extracts, and the number of non-adherent cells (% non-adherence) was related to the protein content of the extract. For this reason it is essential in the tube LAI assay carefully to titrate the different tumour extracts and use a protein concentration of each tumour extract that induces a similar number of cells from control subjects to be non-adherent, in the range of 40–60 (20–30%) non-adherent cells. In control subjects tumour extracts at a protein concentration of 75 to 150 $\mu$g produced this effect. In melanoma patients the difference in leucocyte non-adherence to the 2 tumour extracts was also optimal at these protein concentrations.

The clear difference between patients with malignant melanoma and control donors, with or without other malignant diseases, in the LAI by malignant melanoma extract, strongly suggests that the tube LAI assay detects a tumour antigen of malignant melanoma. Conversely, the patients with malignant melanoma did not respond to unrelated tumour extracts, including those of breast, ovary, lung and bladder cancers.

Malignant melanoma patients react both to allogeneic and autologous malignant melanoma extracts, and this indicates that malignant melanoma shares a common cross-reacting tumour antigen. Although the present study has not confirmed the presence of tumour-specific transplantation antigens in individual tumour extracts, they cannot be excluded. Our studies on the isolation of the malignant melanoma antigen indicate that the cross-reacting antigen detected by the tube LAI assay is on the cell surface membrane (unpublished observations). By contrast Lewis *et al.* (1969) showed, by indirect membrane immunofluorescence, that serum from melanoma patients reacted against the autologous melanoma cell surfaces and lacked significant cross-reactivity with allogeneic melanoma. Bodurtha *et al.* (1975), by a complement-dependent cytotoxicity assay for antibodies to malignant melanoma, confirmed the findings of Lewis *et al.* (1969). Other investigators, however, have shown cross-reactivity with membrane immunofluorescence and cell-mediated assays (Morton *et al.*, 1968; Currie *et al.*, 1971; Nairn *et al.*, 1972; Hellström *et al.*, 1973; and Hollinshead *et al.*, 1974).

In the present study, it was shown that serum from patients whose leucocytes were reactive in the assay could "arm" normal PBL to the appropriate tumour extract. The serum factor was IgG antibody. Moreover, in other studies Grosser *et al.* (1976) have shown that the reactive cell is phagocytic, glass-adherent in the presence of serum and absence of antigen, and has Fc cell-surface receptors. Hence the LAI phenomenon is mediated by peripheral blood monocytes "armed" with cytophilic anti-tumour antibody.

The results of the present study indicate that the anti-tumour immune response of the malignant melanoma patient was dependent on the extent of the cancer, whereas Halliday *et al.* (1975) have not reported any variation in LAI with extent of disease. Patients with cancer confined to the primary site had strong responses and 11 out of 13 patients had a positive NAI. Patients with regional lymph node involvement (Stage II) appeared to have an equally intense and, in a few instances, even more intense LAI. Patients with widely disseminated melanoma to the viscera had a markedly impaired anti-tumour immune response and 3 out of 5 patients had a negative NAI. One of the 2 reactive patients with Stage III cancer initially had a minimal tumour burden and, as the tumour burden increased, showed a loss of reactivity. By other *in vitro* assays of CMI or humoral immunity, a diminution of tumour-specific immunity in melanoma patients with disseminated disease has been reported (Morton *et al.*, 1968; Lewis *et al.*, 1969; Currie and Basham, 1972; Cochran *et al.*, 1973; Hellström *et al.*, 1973; Heppner *et al.*, 1973; and Unsgaard and O'Toole, 1975).

In this study, LAI was observed to be depressed in malignant melanoma patients with a large tumour burden involving their viscera. Furthermore, by monitoring the reactivity of PBL of malignant melanoma patients, it was observed that leucocyte response diminished as the tumour burden increased. The lack of LAI in patients with large tumour burdens is similar to the observed depression of *in vivo* anti-tumour immunity in experimental animal tumours with increasing tumour burdens (Barski and Youn, 1969; Coggin *et al.*, 1974). This suggests that the LAI assay in test tubes may closely reflect the *in vivo* anti-tumour immune status.

A hypothesis, based on an experimental animal tumour model, is that the release of soluble tumour antigen locally and systemically abrogates *in vivo* the effector arm of the tumour immune response and results in depressed *in vitro* measurements of anti-tumour immunity (Thomson, Eccles and Alexander, 1973; Thomson *et al.*, 1973; Coggin *et al.*, 1974). We suggested that soluble tumour antigen in the microenvironment of the tumour prevents rejection (Thomson, 1975) and, as the tumour load increases, sufficient tumour antigen is released systemically to neutralize systemic anti-tumour immunity. In the present study, this hypothesis was supported by the finding that particulate or soluble melanoma antigen was present in the tissue fluids surrounding peritoneal metastasis.

Melanoma patients had their LAI monitored before and after surgical excision of their tumour. LAI was profoundly depressed during the first 2 weeks after surgery. In a previous paper, we reported the depression of LAI after operation in breast cancer patients (Grosser and Thomson, 1975). Slade *et al.* (1975) showed that many aspects of the immune system are depressed by surgery, and Cochran, John and Gothoskar (1972) reported that all melanoma patients had reduced specific tumour immunity post-operatively as measured by macrophage migration inhibition, and reactivity returned in most cases 6–22 days after operation. Furthermore in this study chemotherapy with DTIC also depressed the response of leucocytes in the LAI assay.

The marked immunodepression of anti-tumour immunity after surgery may be a critical factor either in the establishment or survival of metastases. At the time of surgery, investigators have observed circulating tumour cells in some patients, but no difference in survival rates was recorded between patients with or without metastatic circulating tumour cells (Engell, 1959; Griffiths *et al.*, 1973). In an experimental animal tumour model, Eccles and Alexander (1975) have shown that immunosuppressive therapy up to one month after surgical removal of the primary tumour can increase the number of metastases. Hence, animals seemingly cured surgically carry dormant tumour cells that manifest themselves only after treatments that are immunosuppressive. Circulating tumour cells and/or metastatic microfoci of tumour appear to be in a delicate state of balance between survival and rejection, and the depression of anti-tumour defences by surgery may affect the host's capacity to destroy residual tumour cells.

Most melanoma patients had a return of LAI 1–3 months after surgical excision of their tumour. Tumour-free melanoma patients tested at intervals showed a decline in their anti-tumour immunity 5–6 months post-surgery, and all had a low or negative NAI by the 8th post-surgical month. Among 8 tumour-free individuals who were more than a year post-operative, 6 out of 8 tested had a NAI of 30 or less. Although their mean NAI of 22 was below the cut-off value of 30, it was higher than the mean NAI of the control subjects. With their assay Maluish and Halliday (1974) and Halliday *et al.* (1975) have noted no change in LAI in " cancer-free " patients during the year after tumour excision. In the micro-cytotoxicity assay, O'Toole *et al.* (1973) and Unsgaard and O'Toole (1975) observed that removal of tumours by surgery resulted in a loss of detectable CMI in tumour-free patients even one month after surgery, whereas Hellström *et al.* (1973) reported that the majority of clinically cured patients had tumour-specific CMI during a 1–2-year observation period after surgery.

Although LAI diminished after 6–8 months in tumour-free patients, the pattern of LAI in those patients who eventually manifested recurrent cancer showed 2 different patterns. In one instance, LAI remained elevated 11 months after " curative " surgery and fell shortly before clinical evidence of widespread visceral metastasis. The other pattern showed a fall in LAI, similar to that in patients who remained cancer-free, with a rise in LAI slightly before local or limited visceral recurrence.

Holan *et al.* (1974) and Grosser and Thomson (1975) reported that the addition of serum directly in the tube LAI assay either had a non-specific effect or no effect at all. By contrast, Halliday *et al.* (1975) and Maluish and Halliday (1974) have reported that serum from early and late tumour-bearers " blocks " the assay, and serum from " cured " patients is frequently " unblocking ". We have found that, by pre-incubation of normal leucocytes with serum from reactive patients with melanoma or breast cancer, and washing the cells before plating them in the tube LAI assay, immunologically specific " arming " can be detected. These results are different from those of Halliday *et al.* (1975) in that serum from patients reactive in the assay can " arm " normal leucocytes, and serum from metastatic unreactive patients does not " arm ". Moreover, serum from non-reactive melanoma patients with large tumour burdens can " block " the reactivity of leucocytes from reactive melanoma patients in the tube LAI assay (Grosser and Thomson, 1976). The blocking is immunologically specific and therefore indicates that free antigenic determinants must produce the effect, since immune complexes bind to lymphocytes and monocytes non-specifically.

The LAI assays performed on haemocytometers (Halliday and Miller, 1972)

and in test tubes (Holan *et al.*, 1974; Grosser and Thomson, 1975) appear to differ only methodologically; nevertheless, important differences in the results are observed. Holan (1975) has also observed a weaker LAI in rats with large progressively growing tumours than in rats with small regressing tumours. The explanation for the difference in results is not readily apparent but may be related to the difference in incubation conditions.

The ease and rapidity of performing the tube LAI assay and its reproducibility make it useful to monitor the anti-tumour immune response. The changes observed in PBL reactivity in the LAI assay have diagnostic potential when correlated with detailed clinical knowledge of the patient. Moreover, since the changes in LAI reflect the patient's clinical status, the need for performing blocking and unblocking studies is less necessary. This is important, since a constant source of reactive leucocytes is frequently neither available nor practical.

The authors are grateful to Dr S. O. Freedman, Director of the Division of Immunology, where this work was carried out; to Dr W. P. Duguid, Pathologist-in-Chief; and Drs J. K. MacFarlane, H. C. Brown, H. B. Williams, J. D. Palmer and A. G. Thompson of the Department of Surgery.

We thank J. Weatherhead and B. Charland for their expert technical assistance, and Norma Armstrong for preparing this manuscript.

### REFERENCES

BARSKI, G. & YOUN, J. K. (1969) Evolution of Cell-Mediated Immunity in Mice Bearing an Antigenic Tumour—Influence of Tumour Growth and Surgical Removal. *J. natn. Cancer Inst.*, **43**, 111.

BODURTHA, A. J., CHEE, D. O., LAUCIUS, J. F., MASTRANGELO, M. J. & PREHN, R. T. (1975) Clinical and Immunological Significance of Human Melanoma Cytotoxic Antibody. *Cancer Research*, **35**, 189.

BOYUM, A. (1968) Separation of Leukocytes from Blood and Bone Marrow. *Scand. J. clin. Lab. Invest.*, Supplement, **97**, 21.

COCHRAN, A. J., JEHN, V. W. & GOTHOSKAR, B. P. (1972) Cell-Mediated Immunity in Malignant Melanoma. *Lancet*, i, 1340.

COCHRAN, A. J., MACKIE, R. M., THOMAS, C. E., GRANT, R. N., CAMERON-MOWAT, D. E. & SPILG, W. G. S. (1973) Cellular Immunity to Breast Carcinoma and Malignant Melanoma. *Br. J. Cancer*, **28**, Suppl. 1, 77.

COCHRAN, A. J., SPILG, W. G. S., MACKIE, R. M. & THOMAS, C. E. (1972) Postoperative Depression of Tumour-directed Cell-mediated Immunity in Patients with Malignant Disease. *Br. med. J.*, iv, 67.

COGGIN, J. H., AMBROSE, K. R., DIERLAM, P. J. & ANDERSON, N. G. (1974) Proposed Mechanisms by which Autochthonous Neoplasms Escape Immune Rejection. *Cancer Research*, **34**, 2092.

CURRIE, G. A. & BASHAM, C. (1972) Serum Mediated Inhibition of the Immunological Reactions of the Patient to his own Tumour: A Possible Role for Circulating Antigen. *Br. J. Cancer*, **26**, 427.

CURRIE, G. A., LEJEUNE, F. & FAIRLEY, G. H. (1971) Immunization with Irradiated Tumour Cells and Specific Lymphocytotoxicity in Malignant Melanoma. *Br. med. J.*, ii, 305.

DEVRIES, J. E., RUMKE, P. & BERNHEIM, J. L. (1972) Cytotoxic Lymphocytes in Melanoma Patients. *Int. J. Cancer*, **9**, 567.

ECCLES, S. A. & ALEXANDER, P. (1975) Immunologically-mediated Restraint of Latent Tumour Metastasis. *Nature, Lond.*, **257**, 52.

ENGELL, H. C. (1959) Cancer Cells in the Blood—A 5 to 9 Year Follow-up Study. *Ann. of Surgery*, **149**, 457.

FOSSATI, G., COLNAGHI, M. I., DELLA PORTA, G., CASCINELLI, N. & VERONSESI, V. (1971) Cellular and Humoral Immunity against Malignant Melanoma. *Int. J. Cancer*, **8**, 344.

GRIFFITHS, J. D., MCKINNA, J. A., ROWBOTHAM, H. D., TSOLAKIDIS, P. & SALSBURY, A. J. (1973) Carcinoma of the Colon and Rectum; Circulating Malignant Cells and 5-year Survival. *Cancer, N.Y.* **31**, 226.

GROSSER, N., MARTI, J. H., PROCTOR, J. W. & THOMSON, D. M. P. (1976) Tube Leukocyte Adherence Inhibition Assay for the Detection of Anti-tumour Immunity. I. Monocyte is the Reactive Cell. *Int. J. Cancer* (in press).

GROSSER, N. & THOMSON, D. M. P. (1975) Cell-Mediated Anti-tumour Immunity in Breast Cancer Patients Evaluated by Antigen-induced Leukocyte Adherence Inhibition in Test Tubes. *Cancer Research*, **35**, 2571.

GROSSER, N. & THOMSON, D. M. P. (1976) Tube Leukocyte (Monocyte) Adherence Inhibition Assay for the Detection of Anti-tumour Immunity. III. "Blockade" of Monocyte Reactivity by Excess Free Antigen and Immune Complexes in Advanced Cancer Patients. *Int. J. Cancer* (in press).

HALLIDAY, W. J., MALUISH, A. & ISBISTER, W. H. (1974) Detection of Anti-tumour Cell Mediated Immunity and Serum Blocking Factors in Cancer Patients by the Leukocyte Adherence Inhibition Test. *Br. J. Cancer*, **29**, 31.

HALLIDAY, W. J., MALUISH, A., LITTLE, J. H. & DAVIS, N. C. (1975) Leukocyte Adherence Inhibition and Specific Immunoreactivity in Malignant Melanoma. *Int. J. Cancer*, **16**, 645.

HALLIDAY, W. J. & MILLER, S. (1972) Leukocyte Adherence Inhibition: A Simple Test for Cell-mediated Tumour Immunity and Serum Blocking Factors. *Int. J. Cancer*, **9**, 477.

HELLSTRÖM, I. & HELLSTRÖM, K. E. (1973) Some Recent Studies on Cellular Immunity to Melanomas. *Fedn Proc.*, **32**, 156.

HELLSTRÖM, I., WARNER, G. A., HELLSTRÖM, K. E. & SJÖGREN, H. O. (1973) Sequential Studies on Cell-mediated Tumour Immunity and Blocking Serum Activity in 10 Patients with Malignant Melanoma. *Int. J. Cancer*, **11**, 280.

HEPPNER, G. H., STOLBACK, L., BYRNE, M., CUMMINGS, F. J., MCDONOUGH, E. & CALABRESI, P. (1973) Cell-mediated Immunity to Tumour Antigens in Patients with Malignant Melanoma. *Int. J. Cancer*, **11**, 245.

HOLAN, V. (Nov. 1975) Personal communication.

HOLAN, V., HASEK, M., BUBENIK, J. & CHUTNA, J. (1974) Antigen-mediated Macrophage Adherence Inhibition. *Cell Immunol.* **13**, 107.

HOLLINSHEAD, A. C., HERBERMAN, R. D., JAFFURS, W. J., ALPERT, L. K., MINTON, J. P. & HARRIS, J. E. (1974) Soluble Membrane Antigens of Human Malignant Melanoma Cells. *Cancer, N.Y.*, **34**, 1235.

KOLLER, C. A., KING, G. W., HURTUBISE, P. E., SAYNE, A. L. & LOBRIGLIO, A. F. (1973) Characterization of Glass Adherent Human Mononuclear Cells. *J. Immunology*, **iii**, 1610.

LEWIS, M. G., IKONOPISOV, R. L., NAIRN, R. C., PHILLIPS, T. M., HAMILTON-FAIRLEY, G., BODENHAM, D. C. & ALEXANDER, P. (1969) Tumour Specific Antibodies in Human Malignant Melanoma and their Relationship to Extent of Disease. *Br. med. J.*, iii, 547.

LOWRY, O. H., ROSENBROUGH, N. J., FARR, A. L. & RANDALL, R. J. (1963) Protein Measurement with the Folin Phenol Reagent. *J. biol. Chem.*, **193**, 265.

MALUISH, A. & HALLIDAY, W. J. (1974) Cell-mediated Immunity and Specific Serum Factors in Human Cancer: The Leukocyte Adherence Inhibition Test. *J. natn. Cancer Inst.*, **52**, 1415.

MORTON, D. L., EILBER, F. R. & MALMGREN, R. A. (1971) Immune Factors in Human Cancer: Malignant Melanomas, Skeletal and Soft Tissue Sarcomas. *Prog. exp. Tumour Res.*, **14**, 25.

MORTON, D. L., MALGREN, R. A., HOLMES, E. C. & KETCHAM, A. S. (1968) Demonstration of Antibodies Against Human Malignant Melanoma by Immunofluorescence. *Surgery*, **65**, 223.

NAIRN, R. C., NIND, A. P., GULI, E. P., DAVIES, D. J., LITTLE, J. H., DAVIS, N. C. & WHITHEAD, R. H. (1972) Anti-tumour Immunoreactivity in Patients with Malignant Melanoma. *Med. J. Australia*, **1**, 397.

OREN, M. E. & HERBERMAN, R. B. (1971) Delayed Cutaneous Hypersensitivity Reactions to Membrane Extracts of Human Tumour Cells. *Clin. exp. Immunol.*, **9**, 45.

O'TOOLE, C., UNSGAARD, B., ALMGARD, L. E. & JOHANSSON, B. (1973) The Cellular Immune Response to Carcinoma of the Urinary Bladder: Correlation to Clinical Stage and Treatment. *Br. J. Cancer*, **28**, Supplement, 1, 266.

REIF, A. E. (1969) Batch Preparation of Rabbit G-globulin with DEAE Cellulose. *Immunochemistry*. **6**, 723.

SLADE, M. S., SIMMONS, R. L., YUNIS, E. & GREENBERG, L. J. (1975) Immunodepression after Major Surgery in Normal Patients. *Surgery, St Louis*, **78**, 363.

THOMSON, D. M. P. (1975) The Presence of Soluble Tumour-specific Antigen and its Relationship to Tumour Growth. *Int. J. Cancer*, **15**, 1016.

THOMSON, D. M. P., ECCLES, S. & ALEXANDER, P. (1973) Antibodies and Soluble Tumour-specific Antigens in Blood and Lymph of Rats with Chemically Induced Sarcomata. *Br. J. Cancer*, **28**, 6.

THOMSON, D. M. P., SELLENS, V., ECCLES, S. & ALEXANDER, P. (1973) Radioimmunoassay of Tumour Specific Transplantation Antigen of a Chemically Induced Rat Sarcoma: Circulating Soluble Tumour Antigen in Tumour Bearers. *Br. J. Cancer*, **28**, 377.

UNSGAARD, B. & O'TOOLE, C. (1975) The Influence of Tumour Burden and Therapy on Cellular Cytotoxicity Responses in Patients with Ocular and Skin Melanoma. *Br. J. Cancer*, **31**, 301.